# Biodiscovery of Actinomycetota through metabolo-genomics reveals functional diversity across contrasting Mexican ecosystems

**DOI:** 10.1099/mgen.0.001557

**Published:** 2025-10-31

**Authors:** Lorena Rodríguez-Orduña, César Aguilar, Alan Gerardo Hernández-Melgar, Héctor F. Arocha Garza, Karina Verdel-Aranda, Augusto Vázquez Rodríguez, José L. López-Ribot, Aldo Moreno-Ulloa, Cuauhtémoc Licona-Cassani

**Affiliations:** 1 Tecnológico de Monterrey, Industrial Genomics Laboratory, Centro de Biotecnología FEMSA, Escuela de Ingeniería y Ciencias, Monterrey, N.L., Mexico; 2 Biomedical Innovation Department, CICESE, Ensenada, B.C., Mexico; 3 Genesis Laboratory, Cuatro Ciénegas de Carranza, Coahuila, Mexico; 4 Tecnológico Nacional de México, Instituto Tecnológico de Chiná, Chiná, Campeche, Mexico; 5 Department of Molecular Microbiology and Immunology and South Texas Center for Emerging Infectious Diseases, The University of Texas at San Antonio, San Antonio, Texas, USA; 6 Instituto de Biotecnología, Universidad Nacional Autónoma de México, Cuernavaca, Morelos, 62210, Mexico; 7 Tecnológico de Monterrey, Integrative Biology Research Unit, The Institute for Obesity Research, Monterrey, N.L., Mexico

**Keywords:** *Actinomycetota*, genome mining, metabolo-genomics, natural products, *Nocardia*, *Streptomyces*

## Abstract

*A corrigendum of this article has been published; full details can be found at [10.1099/mgen.0.001641]*

Discovery of novel bioactive natural products from *Actinomycetota* has decreased, motivating the exploration of rare ecosystems. In this work, we integrated genomics and untargeted metabolomics to profile the biosynthetic capacity of *Streptomyces* and *Nocardia* strains isolated from two contrasting, Ramsar-listed Mexican biomes with well-defined ecological features: Cuatro Ciénegas, a semi-arid, oligotrophic karst basin with high microbial endemism, and Calakmul, a tropical rainforest characterized by high biodiversity, seasonal wetlands and limestone-derived alkaline soils. We hypothesized that bacteria isolated from contrasting environments would encode for novel and distinctive subsets of secondary metabolites. Using different metabolo-genomic approaches, a diverse collection of *Streptomyces* and *Nocardia* strains was identified, including several potentially novel species. Genome mining revealed a large repertoire of biosynthetic gene clusters (BGCs), many without matches to known metabolites, while molecular networking and dereplication (GNPS, SNAP-MS) exposed extensive chemical diversity within the isolates. Targeted synteny/ortholog analyses (CORASON) linked subsets of metabolites to BGCs, confirming actinomycin and collismycin and identifying komodoquinone-, nocardiopsistin- and rubiginone-like clusters. Notably, bioactivity assays of crude extracts demonstrated effective antifungal effects against *Candida albicans* SC5314 biofilms and planktonic growth, suggesting their potential therapeutic use. These findings reveal a significant untapped chemical space encoded by *Actinomycetota* from Ramsar sites, while reinforcing the need for improved tools to connect genomic and metabolomic data for natural product discovery.

**Impact Statement**

Our study highlights the relevance of *Streptomyces* and *Nocardia* strains isolated from contrasting Mexican ecosystems, including the semiarid soils of Cuatro Ciénegas and the tropical forest of Calakmul. We present a metabolo-genomic dataset for 18 isolates grown under standardized laboratory conditions and identify persistent challenges in linking biosynthetic gene clusters (BGCs) to their metabolites with current methods. Conceptually, we demonstrate that ecological context imprints biosynthetic repertoires, supporting targeted bioprospecting in diverse niches. These advances enhance genome–metabolome linkage, prioritize promising strains and BGCs and accelerate translation toward new anti-infective compounds, while underscoring the value of conserving biodiversity-rich wetlands as reservoirs of chemical diversity.

## Data Summary

The raw LC-MS² datasets were submitted to the GNPS/MassIVE public repository under accession number MSV000097984 (https://massive.ucsd.edu/ProteoSAFe/dataset.jsp?task=8ff83c737e0b4786b377cbfc753a348b), accessed on 23 May 2025. Data analysis was carried out using several GNPS workflows, including Feature-Based Molecular Networking (https://gnps.ucsd.edu/ProteoSAFe/status.jsp?task=6144d573bc4a40f4bc325b21519d7353, accessed on 1 October 2024), DEREPLICATOR+ (https://gnps.ucsd.edu/ProteoSAFe/status.jsp?task=b7af447cb38d407f8d6e4bd667590d60, accessed on 2 October 2024) and MOLDISCOVERY (https://gnps.ucsd.edu/ProteoSAFe/status.jsp?task=691e043b641e4d119ce140c4e4d3c40b, accessed on 2 October 2024).

## Introduction

Natural products (NPs) have inspired the development of bioactive molecules with applications in human health and agriculture, including antibiotics, antifungals, anticancer agents, immunosuppressants and crop protection compounds [1]. Micro-organisms are a particularly rich reservoir of NPs, offering a scalable source of chemically diverse molecules. Among bacteria, *Actinomycetota* are especially prolific NP producers, contributing to nearly half of all described microbial natural products, including more than 10,000 antimicrobial compounds [2,3]. Efforts to discover novel natural products have prioritized the study of metabolites in microbes isolated from rare (or poorly explored) ecosystems, recognizing the central role of secondary metabolism in conferring ecological fitness to the host.

Rare actinomycete families or well-studied *Streptomyces* species isolated from rare environments (e.g. environments shaped by physicochemical stressors that impose unique selective pressures) are particularly enriched in novel natural products [4,6]. Under highly oligotrophic or other extreme environmental conditions, secondary metabolism provides the host with a toolkit of chemically diverse metabolites for adaptive or survival purposes. For instance, drought-adapted isolates from the Atacama Desert are prolific producers of atacamycins, a family of 22-member macrolactone metabolites with antibiotic activity [7]. Other examples are reported for isolates from deep-sea sediments that produce a wide range of structurally unique metabolites with pharmaceutical potential [8].

Wetlands recognized under the Ramsar Convention are particularly interesting for microbiologists due to their exceptional environmental conditions, including karstic, oligotrophic and intermittently flooded systems [9]. In Mexico, the Cuatro Ciénegas Basin (CCB) and the Calakmul Biosphere Reserve (CBR) represent the most prominent and contrasting Ramsar sites. The CCB, located in the Chihuahuan Desert, is characterized by extreme nutrient limitation, calcium-rich sediments and a nitrogen-to-phosphorus ratio reminiscent of ancient oceans [10]. In contrast, the tropical CBR rainforest, with its shallow alkaline soils over limestone and seasonal wetlands, represents a nutrient-fluctuating environment with high potential for isolating microbes and identifying novel NPs [11].

Different omics approaches are widely used to prioritize microbial isolates for natural product discovery [12,14]. Genome mining tools enable the systematic identification of biosynthetic gene clusters (BGCs), while metabolomics allows the characterization and rapid dereplication of molecules [15]. While several integrative workflows have accelerated the field of NP discovery [16,17], establishing robust BGC-to-NP linking pipelines remains a major challenge. The high prevalence of cryptic/silent BGCs in environmental strains requires extensive manual curation in metabolo-genomics datasets. Despite these limitations, the integration of genomics, metabolomics and computational biology provides an unprecedented opportunity to uncover the hidden biosynthetic capacity of actinomycetes from extreme environments [14].

Here, we present the identification and characterization of 18 bacterial strains isolated from two contrasting Ramsar-listed wetlands in Mexico. We characterized the biosynthetic potential for all the environmental isolates using antiSMASH and BiG-SCAPE and cross-linked these predictions with untargeted metabolomics datasets generated from different standard culture media. Exhaustive manual curation allowed us to correlate several natural products to BGCs. Our results show that the Cuatro Ciénegas Basin and the Calakmul Biosphere Reserve are ecological niches that harbor strains with high potential for natural product discovery. More importantly, our results provide for the first time a high-quality structured metabolo-genomics dataset that can be used to complement other approaches for the prediction and prioritization of novel biosynthetic gene clusters and their associated metabolites.

## Methods

### Sample collection and strain isolation

Soil samples were collected from the Churince lagoon in Cuatro Ciénegas Basin, Coahuila (26.84391, –102.12954) during October 2018 and the Calakmul forest in Campeche (18.89557, −89.38478) during June 2019. To maintain the integrity of the collected samples, they were immediately transferred into sterile plastic tubes and stored at 4 °C until further processing. In the laboratory, all samples were resuspended in phosphate buffer solution (PBS) in serial dilutions from 10^−1^ to 10^−5^ [18]. Aliquots of 100 µl were plated using glass beads on solid media plates and incubated at 30 °C for 5–30 days. We used culture media, including ISP2 (glucose, 0.4 g; yeast extract, 0.4 g; malt extract, 1 g; agar, 18 g; distilled water, 1 l), SCA (soluble starch, 10 g; K_2_HPO_4_, 2 g; KNO_3_, 2 g; casein, 0.3 g; MgSO_4_.7H_2_O, 0.05 g; CaCO_3_, 0.02 g; FeSO_4_.7H_2_O, 0.01 g; agar, 15 g; and filtered sea water, 1 l; pH, 7.0 ± 0.1), oligotrophic (peptone, 1 g; yeast extract, 0.5 g; K_2_HPO_4_ H_2_O 1 g, MgSO_4_ 7H_2_O, 0.5 g; CaCO_3_ 0.3 g, NaCl 5 g; vitamin mix; agar, 15 g; distilled water 1 l) and YIM 212 (histidine, 1 g; raffinose, 5 g; K_2_HPO_4_ 3H_2_O, 1 g; MgSO_4_ 7H_2_O, 0.5 g; agar 20 g; distilled water 1 l). All media were supplemented with cycloheximide to inhibit fungal growth and nalidixic acid to inhibit the growth of Gram-negative bacteria. Microbial colonies exhibiting characteristic *Actinomycetota* morphology, such as powdery or chalky appearance, filamentous growth, branching hyphae and pigmented spores were selectively isolated through a streak until axenic cultures were obtained. All isolates were stored in glycerol stocks at −80 °C until further analysis.

### Genome sequencing and assembly

DNA was extracted from 50 ml liquid cultures of ISP2 media (in 250 ml flasks), incubated at 30 °C, 200 r.p.m. for 2-3 days. For each isolate, biomass pellets were obtained through centrifugation (10,000 r.p.m., 10 min, 4 °C). Cell lysis was performed by pulverizing the biomass with liquid nitrogen in a mortar. Chemical lysis was performed by adding buffer lysis solution together with 20 µl of lysozyme (20 mg ml^−1^) and incubating for 1 h at 37 °C, followed by adding 20 µl of proteinase K (0.2 mg ml^−1^) and 20 µl of 10% SDS with 40 min incubation at 55 °C. DNA extraction was performed with phenol/chloroform/isoamyl alcohol (25:24:1) standard protocol, recovering the aqueous phase and precipitating the DNA with ethanol and 3M sodium acetate. The resultant pellet was collected by centrifugation, washed with 70% ethanol and dissolved in deionized water. The quality and yield of the obtained DNA were assessed on a NanoDrop spectrophotometer and agarose gel electrophoresis. For whole-genome sequencing, genomic libraries were prepared using the Illumina DNA Prep Kit with IDT 10 bp indices and sequenced on an Illumina NextSeq 2000 platform, obtaining paired-end reads of 2×151 bp. In addition, long reads were obtained by Oxford Nanopore Technologies. First, the preparation of libraries was performed with the PCR-free Oxford Nanopore Technologies ligation sequencing kit without additional DNA fragmentation. The sequencing was performed on an Oxford Nanopore MinION Mk1B sequencer using R10.4.1 flow cells. We used the MinKNOW GUI 5.3.6 platform with a minimum read length of 200 bp and the default parameters. Genome assemblies were obtained by *de novo* assembling in the Bacterial and Viral Bioinformatics Resource Center [19] service using the Unicycler assembler [20], followed by genome annotation on the Rapid Annotation using Subsystems Technology platform [21].

### Taxonomic and phylogenomic analysis

All genome assemblies were submitted to Microbial Genomes Atlas version 1.3.21.5 available at https://uibk.microbial-genomes.org/ [22] to obtain an approximation of their taxonomic classification, using the TypeMat database to obtain Average Nucleotide Identity (ANI) values [23]. ANI values were calculated as pairwise genome similarity based on whole-genome alignments. A threshold of 95% ANI was used to determine whether strains belonged to the same species. For strains that did not yield ANI results in the MIGA platform, the EZBioCloud tool (https://www.ezbiocloud.net/tools/ani) was used, selecting as a reference the genome with the highest digital DNA–DNA hybridization (dDDH) value according to Type (Strain) Genome Server (TYGS) results. Finally, to establish evolutionary relationships and give a more comprehensive taxonomic framework, phylogenomic analysis was performed using the TYGS, accessible at https://tygs.dsmz.de/ [24]. The phylogenomic tree (whole-genome sequenced-based) obtained was edited with isolates metadata, including isolation site, BGCs class content and biosynthetic novelty index (BiNI) score.

### BGC annotation

Genome mining was performed in the antiSMASH 8.0 server available at https://antismash.secondarymetabolites.org/#!/start using as parameters ‘relaxed strictness’ and ‘all features’ on [25]. Complete BGCs were selected for the construction of the BiG-SCAPE [26] cluster-type sequence similarity network. Clusters labeled as ‘contig edge’ by antiSMASH were considered incomplete and excluded from this analysis, whereas clusters containing the core biosynthetic genes without this label were considered as complete BGCs. For the network construction, gene clusters of the MIBiG database [27] were used, and singletons were included. A similarity score threshold of 0.3 was applied in BiG-SCAPE to determine significant matches between the BGCs reported in this study and MIBiG references. The network was visualized in Cytoscape 3.9.0 [28] according to the isolation site and taxonomic classification or the BGC class prediction. BiNI [5] was calculated as the average of the distances (resulting in BiGFAM analysis) of all BGCs contained in each strain, to know the novelty of their biosynthetic gene cluster content. The relationship between genome size and the proportion of incomplete BGCs was modelled using a linear regression.

### Metabolic extractions

Biomass pellets were obtained for each strain from solid cultures in ISP2 and ISP5 media. For this purpose, the surface of the media was covered with a sterile cellophane disc before inoculation, to recover the biomass without media. The plates were incubated at 30 °C, and the biomass was collected with a spatula in a microtube and lyophilized for 24 h. Lyophilized samples were pulverized using a mixer mill (Retsch MM400) set at 30 m s^−1^ for 35 s. A total of 5 mg of the powder was extracted using 500 µl of solvent mixture (methanol, acetonitrile and ethyl acetate in equal proportions). All extractions were performed under sonication for 30 min at room temperature. After sonication, the samples were centrifuged at 14,000 r.p.m. for 5 min at 4 °C. The resulting supernatants were transferred to 1.5-ml Eppendorf tubes for the evaporation of the solvent using a SpeedVac at room temperature. Once dried, the extracts were weighed and reconstituted to a final concentration of 300 ng µl^−1^ using a water/acetonitrile solution (80:20). The reconstituted extracts were centrifuged at 14,000 r.p.m. for 5 min at 4 °C, and the clear supernatant was collected for subsequent analysis. For quality control (QC) samples, equal volumes of these particle-free supernatants were pooled into a single tube.

### Untargeted LC-MS² data acquisition

We used the instrumentation and equipment settings outlined previously (https://pubs.acs.org/doi/10.1021/acs.jproteome.4c00256) [29]. Metabolite separation was performed by injecting 2 µl of each sample into an Agilent nanoLC 1260 Infinity system (Agilent Technologies, Santa Clara, CA), equipped with a ZORBAX 80 SB-C18 analytical column (75 µm×43 mm, 5 µm particle size) and a 40-nl enrichment column. The mobile phases consisted of water (A) and acetonitrile (B), both containing 0.1% formic acid. A flow rate of 300 nl min^−1^ was applied to the separation column (nano pump), while 2 µl min^−1^ was delivered to the enrichment column (micro pump). The chromatographic gradient began at 5% B, increased linearly to 40% B for over 20 min, then to 100% B in 5 min, maintained 100% B for 5 min, returned to 5% B within 1 min and finally held at 5% B for 9 min to allow column re-equilibration. To prevent carryover, two blank injections (3 µl of mobile phase A/B at 95:5) were run between each experimental sample. The eluent was directed to a 6530 Accurate-Mass Q-TOF mass spectrometer (Agilent Technologies) via an HPLC-Chip Cube MS interface. Metabolite ionization was performed using nanospray in positive mode. MS data acquisition parameters were as follows: capillary voltage, 1850 V; gas temperature, 350 °C; drying gas flow, 5 l min^−1^; skimmer voltage, 65 V; octopole RF, 750 V; fragmentor voltage, 175 V; acquisition rate, 4 spectra/s over a mass range of m/z 110–2000. For MS², the instrument was operated with a narrow isolation window (1.3 m/z), acquisition rate of 3 spectra/s and selection of up to five precursor ions per cycle within a range of m/z 50–2000. Active exclusion was enabled, with a threshold of two spectra and a release time of 0.25 min. Collision energies were applied using a ramped method with a slope of 6 and an offset of 4. Prior to data collection, the instrument was externally calibrated using the ESI-L Low Concentration Tuning Mix (Agilent Technologies) to ensure mass accuracy below 5 p.p.m. for both MS and MS² data. QC samples were injected every three to four experimental samples to monitor instrument performance, and blank signals were regularly evaluated. Sample injections were randomized to minimize batch effects.

### LC-MS² data processing and analysis

The LC-MS² datasets were processed using a workflow based on open-access software and online platforms, comprising three main steps as previously described [29] with some modifications. First, feature detection and alignment across all datasets were carried out using Mzmine [30]. Second, univariate and multivariate statistical analyses, along with hierarchical clustering and heatmap visualization, were conducted via MetaboAnalyst 4.0 (https://www.metaboanalyst.ca) [31]. Third, metabolite annotation at the structural level was performed through different methods. First, at the Global Natural Products Social Molecular Networking (GNPS) platform available at https://gnps.ucsd.edu [32], corresponding to level 2 of the Metabolomics Standards Initiative (MSI), *in silico* annotation tools such as CSI:FingerID [33], DEREPLICATOR+ [34] and MolDiscovery [35] were executed, corresponding to MSI level 3. Additionally, the SIRIUS graphical user interface (version 5.8.5) [36] was used to obtain the chemical classes over NPClassifier [37]. The metabolite annotations obtained by both the GNPS spectral library and *in silico* tools were classified into chemical classes by using the NPClassifier ontology [37]. The results from FBMN, DEREPLICATOR+, CSI:FingerID, MolDiscovery, CANOPUS and NPClassifier were combined based on the previous work published by Osorio-Ramirez (https://www.mdpi.com/2072‑6651/17/4/150) using a custom script derived from the MolNetEnhancer workflow [38], which is openly accessible at https://github.com/froz9/MolNetEnhancerMod. The resulting molecular network was edited in Cytoscape [28], with node colors reflecting the superclass level classification. Certain metabolite annotations generated with *in silico* dereplication tools did not include specific chemical names and instead returned only SMILES strings. To resolve this, we used the SMILES-TO-IUPAC-name translator (STOUT) to convert the SMILES representations into IUPAC names [39].

### Natural product annotation via molecular networking and structural fingerprinting

We used the Structural Similarity Network Annotation Platform for Mass Spectrometry (SNAP-MS) to enhance the annotation of natural products in our dataset [40]. The SNAP-MS approach integrates chemical similarity data from the Natural Products Atlas, allowing the assignment of compound families to molecular subnetworks without relying on experimental or *in silico* reference spectra. By combining SNAP-MS with the molecular network, we refined compound annotations, particularly those involving previously ambiguous or unresolved features.

### Antifungal bioactivity of extracts against *Candida albicans* SC5314

An aliquot of 15 µl from the glycerol stock was inoculated on SFM medium and incubated at 30 °C until sporulation. All cultures were passed to ISP2 agar plates with sterilized cellophane and incubated at 30 °C. After 5–7 days, the cellophane was removed, and the remaining solid medium was transferred to a 50-ml Falcon tube, followed by the addition of 1 volume of ethyl acetate. The tubes were vortexed for 1 h, and ethyl acetate was recovered by centrifugation at 4,000 r.p.m. for 10 min and decanted into a 15-ml Falcon tube. The extracts were incubated at 45 °C for evaporation using a CentriVap concentrator for ~2 h. Dried extracts were stored at −20 °C until further processing.

For antifungal bioactivity assays, dried extracts were solubilized in 100 µl of DMSO, sonicated for 5 min and stored at −80 °C until use. The antifungal bioactivity of the extracts was evaluated using the yeast pathogen *C. albicans* strain SC5314. Frozen glycerol stocks of *C. albicans* SC5314 were used to inoculate yeast extract–peptone–dextrose (YPD) (1% (w/v) yeast extract, 2% (w/v) peptone, and 2% (w/v) dextrose) agar plates. Plates were then incubated at 30 °C for 24 h. Individual colonies were then collected using an inoculation loop to inoculate 25 ml of YPD broth, followed by overnight incubation at 30 °C in an orbital shaker. After the incubation, cells were harvested by centrifugation at 4,000 r.p.m. for 5 min, washed twice with PBS and resuspended in PBS. The cell concentration was determined using a disposable counting chamber.

For the working inoculum, cells were diluted in RPMI-140 medium (without sodium bicarbonate, supplemented with l-glutamine, buffered with 165 mM morpholine propane sulphonic acid, and adjusted to pH 6.9) and then adjusted to the desired concentration according to experimental conditions. The antifungal activity of the extracts was assessed on planktonic cells of *C. albicans* following the Clinical and Laboratory Standards Institute guidelines, with minor modifications [41]. Briefly, twofold serial dilutions of each extract (ranging from 4% to 0.062% v/v) were prepared in 100 µl of RPMI medium per well in a 96-well round-bottom plate. Each well was then inoculated with 100 µl of a standardized inoculum (1×10⁴ cells/ml), leading to final concentrations that halved the compound and cell densities. Plates were sealed with gas-permeable films and incubated at 35 °C for 24 h. At the end of the incubation period, OD₆₂₀ was measured using a microplate reader. Raw absorbance values were normalized to calculate the percentage of inhibition, using positive and negative controls for reference. To standardize the results, percentage inhibition values exceeding 100% or falling below 0% were adjusted to 100% and 0%, respectively. Afterwards, the minimum fungicidal concentration (MFC) of the extracts was determined by spot inoculating 50 µl from wells showing complete visual inhibition onto YPD agar plates. The inoculated plates were incubated at 30 °C and checked after 48 h. The MFC was defined as the lowest concentration that resulted in no colonies or fewer than three colonies on the agar plate [42]. Dose-response experiments were performed in duplicate, with three technical replicates per experiment.

The activity of extracts on biofilm formation was also evaluated on *C. albicans* SC5314 as follows. Serial twofold dilutions of each extract were prepared in RPMI medium (50 µl per well) and dispensed into 96-well flat-bottom cell-culture-treated plates. Each well was then inoculated with 50 µl of a working inoculum containing 2×10⁶ cells ml^−1^, resulting in final extract concentrations ranging from 2% µM to 0.031% (v/v) and a twofold dilution of cell density. Plates were sealed with gas-permeable films and incubated at 37 °C for 24 h to allow biofilm formation. After incubation, the supernatant was carefully removed, and the wells were washed twice with PBS. Biofilm metabolic activity was assessed by adding 100 µl of 2,3-bis(2-methoxy-4-nitro-5-sulfo-phenyl)−2H-tetrazolium-5-carboxanilide (XTT) solution (0.5 mg/ml, supplemented with 2.5 µM menadione) to each well. Plates were incubated for 1 h at 37 °C, and absorbance was measured at 490 nm using a microplate reader. The percentage of inhibition was calculated as previously described. Dose-response experiments were performed in duplicate, with three technical replicates per experiment. All our plate assays included both positive and negative growth controls, with the positive growth controls being wells to which cells are added and allowed to grow uninhibited (cells and media only in the absence of any extract/inhibitor). The negative growth controls are wells to which only media (but no cells) is added. In addition, we also include wells treated with a series of twofold decreasing concentrations of amphotericin B, which represent a positive control for antimicrobial activity.

## Results

### Strain isolation and taxonomic classification

Soil bacteria were isolated from wetlands in the CCB (26.84391, –102.12954) and CBR (18.89557, –89.38478) to study their biosynthetic capacity. We obtained a total of 18 environmental isolates using selective media (SCA [43], ISP2 [44], oligotrophic [45], YIM 212 [46]) and standard culture conditions (see the ‘Methods’ section). For simplicity, strains isolated from Cuatro Ciénegas were labelled with the prefix ‘CC’, and those from Calakmul were labelled with the ‘KL’ prefix.

Genome sequences were obtained for all the strains isolated in this study (Table 1). We identified 14 isolates that belong to the *Streptomyces* genus (9 strains isolated from CCB and 5 isolated from CBR), and 4 strains were identified as *Nocardia* sp*.* (from CCB). Based on ANI values (< 95%), we believe that nine isolates (seven from CCB and two from CBR) could be taxonomically assigned to a novel species. Notably, two *Streptomyces* isolates from CCB (CC210A and CC208B) have even lower ANI values (<80%). We performed 16S-rRNA blast and identified that *Streptomyces coeruleoprunus* is the closest relative (with 99.46% and 99.73% identity, respectively). Further analyses using multilocus sequence type indicated that CC2010A and CC208B are closest to *Streptomyces thermolilacinus*, with ANI values of 86.7% and 86.6%. Table 1 enlists the closest relative strains to all the isolates reported in this study based on the Microbial Genome Atlas taxonomic classification pipeline. A summary of all quality genome assembly statistics can be found in Table S1, available in the online Supplementary Material.

**Table 1.** Genomic and taxonomic characteristics of isolated strains and their biosynthetic potential

Strain	Closest relative	ANI (%)	BiNI	Isolation site	# BGC	Accession no.	Isolation media
KL110B	*Streptomyces hydrogenans NBRC 13475*	98.7	570.5	CBR, shore 21 cm	11	JASERM000000000	SCA
KL118A	*Streptomyces atriruber NRRL B-24165*	97.79	752.5	CBR, soil 21 cm (aguada shore)	11	JASERJ000000000	ISP2
CC216C	*Streptomyces parvus JCM 4069*	97.55	618.1	CCB, Churince well	36	+JASCXQ000000000	ISP2
CC216B	*Streptomyces parvus JCM 4069*	97.54	659.2	CCB, Churince well	19	JASCXK000000000	ISP2
CC213D	*Streptomyces parvus NRRL B-1455*	96.95	805.5	CCB, Churince well	21	JASCXM000000000	SCA
KL122B	*Streptomyces venezuelae ATCC 10712*	96.27	614.6	CBR, centre 15 cm	27	+JASERI000000000	Oligotrophic
CC201C	*Nocardia otitidiscaviarum NCTC1934*	95.89	516	CCB, Churince well	25	+JASCXP000000000	SCA
CC216A	*Nocardia otitidiscaviarum NCTC1934*	95.86	459.4	CCB, Churince well	22	JASFXB000000000	ISP2
CC213A	*Nocardia otitidiscaviarum NCTC1934*	95.84	452	CCB, Churince well	23	JASFXA000000000	SCA
KL109B	^*^Streptomyces roseolus JCM 4411	94.25	848.2	CBR, soil 5 cm (aguada limits)	29	JASERL000000000	SCA
KL111A	^*^Streptomyces roseolus JCM 4411	93.79	760.9	CBR, cycad	20	+JASGLF000000000	YIM 212
CC227C	^*^Nocardia otitidiscaviarum NCTC1934	92.45	597.9	CCB, Churince well	27	JASITE000000000	ISP2
CC208A	^*^Streptomyces roseolus NBRC 12816	91.27	663.4	CCB, Churince well	18	JASCXN000000000	SCA
CC224E	^*^Streptomyces roseolilacinus JCM 4335	83.92	999.4	CCB, Churince well	31	+JASITF000000000	ISP2
CC228A	^*^Streptomyces coeruleoprunus JCM 6919	83.67	657.5	CCB, Churince well	20	JAJPUI000000000	ISP2
CC219A	^*^Streptomyces fradiae DSM 40063	83.22	499.5	CCB, Churince well	11	JAJPUH000000000	SCA
CC210A	^*^Streptomyces fradiae ATCC 10745	78.73	616.3	CCB, Churince well	21	JAJPUG000000000	ISP2
CC208B	^*^Streptomyces fradiae ATCC 10745	70.02	771.9	CCB, Churince well	16	CP045031	ISP2

*Potential novel species according to ANI values (< 95%). +New assembly with higher quality is available upon request to the corresponding author.

### Phylogenomic analysis

**Fig. 1.**
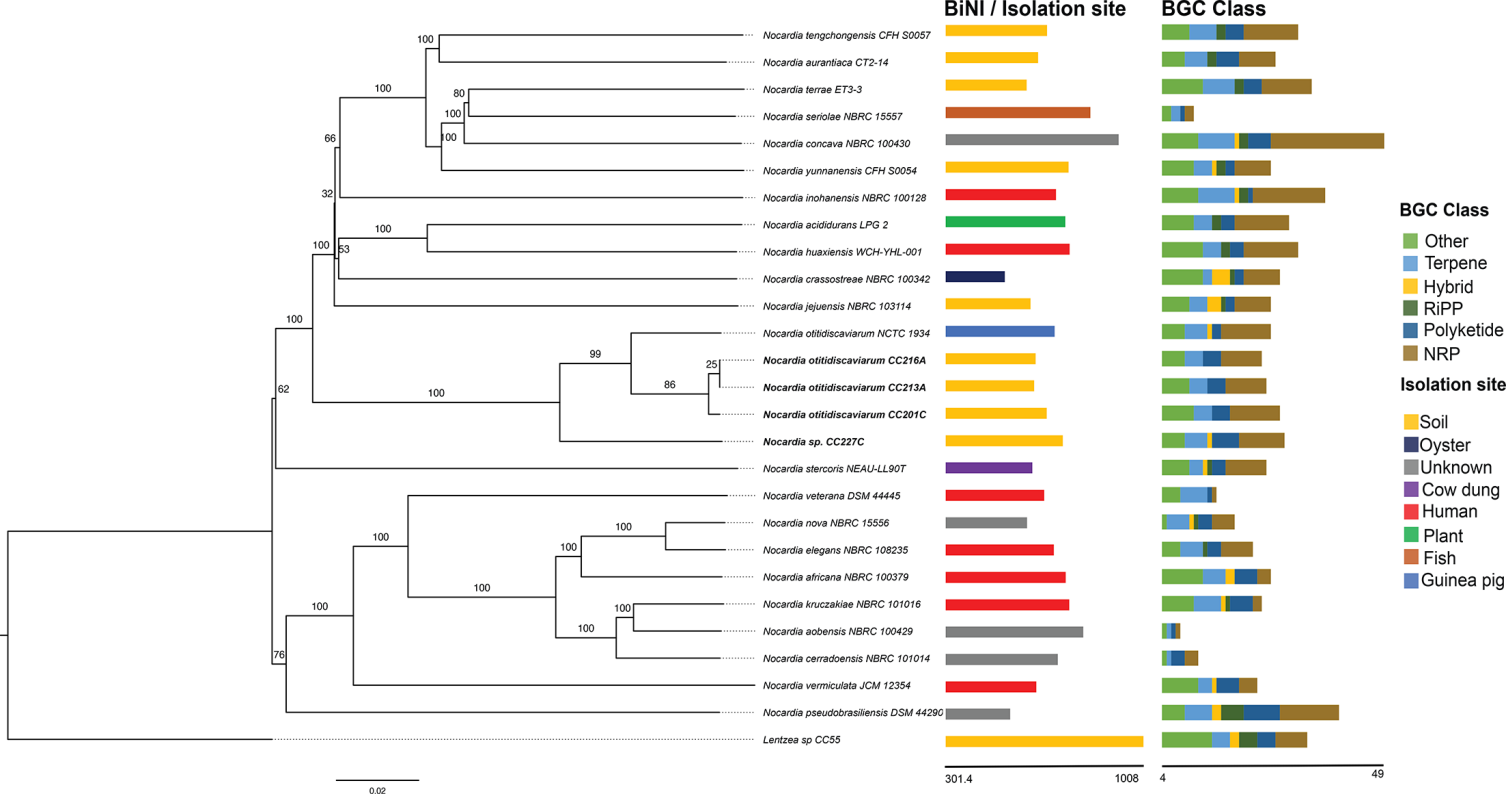
Whole-genome sequence-based phylogenomic tree of *Nocardia* strains. Phylogenetic trees were constructed using TYGS, and branch support values (bootstrap) are indicated at the nodes to show the robustness of the phylogenetic relationships. The first annotation column indicates the total number and class distribution of BGCs identified by antiSMASH. The second column displays the BiNI scores (represented by the bar plot), indicating the degree of novelty in the BGC repertoire for each strain. Isolation source is coded by colour. The name of strains isolated from Cuatro Ciénegas are shown in bold.

**Fig. 2.**
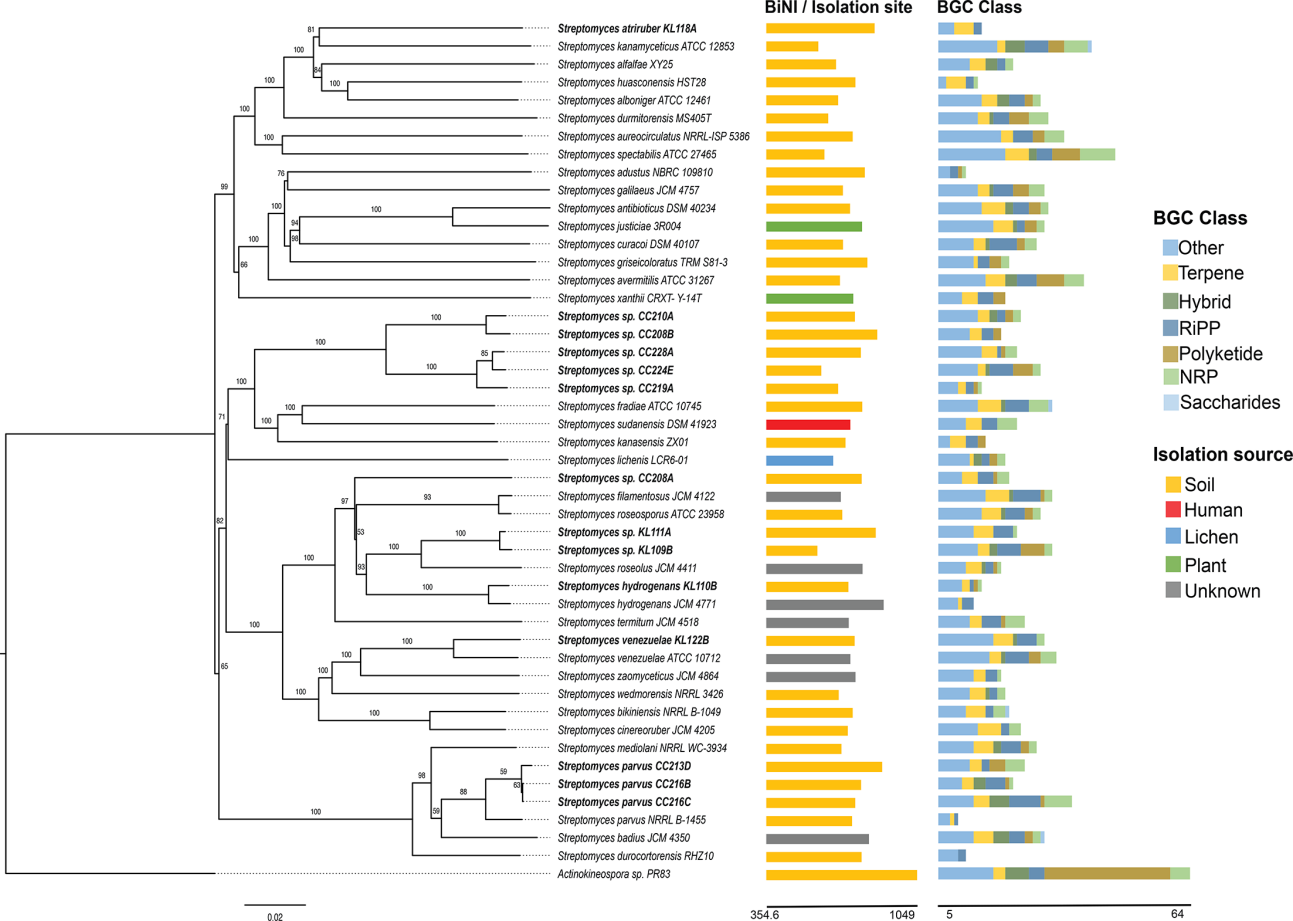
Whole-genome sequence-based phylogenomic tree of *Streptomyces* strains. The tree was generated using TYGS, and bootstrap support values are indicated at the nodes to evaluate the robustness of the phylogenetic relationships. The first annotation column shows the total number and distribution of BGCs classified by type, as predicted by antiSMASH. The second column represents the BiNI scores (bar plots) which indicate the relative novelty of the BGC repertoire for each strain. The isolation source is coded by colour. The names of strains isolated from Cuatro Ciénegas and Calakmul are shown in bold.

In order to understand the evolutionary relationships of the strains in our study, we used the TYGS (https://tygs.dsmz.de/) [24] platform (Figs1  2). TYGS relies on the Genome blast Distance Phylogeny approach, which allows robust tree construction with statistical support for the branches. The phylogenomic analysis of *Streptomyces* isolates revealed four core clades (Fig. 2). Several strains – CC224E, CC228A, CC219A, CC208B and CC210A – formed a monophyletic group, while KL109B and KL111A were also monophyletic, representing the most divergent clade within their sub-branch. Additionally, CC216C, CC216B and CC213D clustered within a distinct core clade alongside *Streptomyces parvus*, a soil-derived species. Isolates CC208A and KL118A appeared as unique taxa within the phylogeny. As expected, the phylogenetic analysis of *Nocardia* confirmed that all our strains clustered within a single clade alongside members of *Nocardia otitidiscaviarum*, marked in blue colour in Fig. 2.

### BGC annotation and novelty analysis

In order to explore the biosynthetic potential of our isolates, we used antiSMASH (version 8.0) and annotated a total of 596 BGCs across all genomes. An expected relationship between the genome quality (completeness) and the portion of complete BGCs per genome was observed. Specifically, a stronger correlation was detected between the number of contigs and the percentage of incomplete BGCs (*R*=0.64). Overall, the contig counts in our dataset range from a closed genome (CC208B, 1 contig) to fragmented assemblies (CC219A, 334 contigs), with a median of 47 contigs. Eleven of the 18 assemblies have <60 contigs, including 4 genome drafts with <20 contigs (CC210A, CC216C, CC224E and KL122B). A total of 6 strains exceed 100 contigs (CC208A, 106; KL111A, 146; CC216B, 187; KL110B, 259; KL118A, 250; CC219A, 334), indicating a higher number of fragmented BGCs in such strains (Table S1).

Next, we assessed the novelty of the annotated BGCs. We applied the BiNI pipeline, which uses BiG-FAM distance matrices to estimate the potential for novel metabolite production [5]. BiNI quantifies how distinct the repertoire of BGCs in a strain’s genome (based on BiG-FAM distance matrices) is relative to known BGCs in public databases. A high BiNi score indicates that a given strain has, on average, a more unique subset of BGCs. The BiNI scores of the 14 *Streptomyces* isolates ranged from ~450 to ~1000, while for *Nocardia* strains, BiNI scores ranged from ~450 to ~600. This indicates that the *Nocardia* strains have a comparatively reduced biosynthetic diversity, reflecting differences in their secondary metabolite repertoire relative to *Streptomyces*.

*Nocardia* strain CC213A showed the lowest score, while *Streptomyces* CC224E showed the highest. Six *Streptomyces* strains showed high predicted novelty (BiNI >750) (KL118A, CC213D, KL109B, KL111A, CC224E and CC208B). Isolates KL109B and CC213D showed particularly high BiNI scores (848.2 and 805, respectively), which are well above the median values typically observed for actinomycetota (<400). Such isolates showed ANI values far below the commonly accepted 95% threshold for species delineation (Table 1). In this context, uniqueness refers to the combination of low genomic similarity with other strains (as measured by ANI) and high biosynthetic distinctiveness (as indicated by BiNI). Conversely, CC224E displayed the highest BiNI score (999.4) while maintaining an ANI value above 97% (Fig. 2 and Table 1).

### BiG-SCAPE cluster type sequence similarity network of BGCs

To assess the biosynthetic potential of our 18 strains, we constructed a BiG-SCAPE sequence similarity network using the MiBIG 3.0 library [47]. The resulting network comprised 632 nodes, categorized into distinct classes such as NRPS (*n*=121), PKSI (*n*=39), others (*n*=226), PKS-other (*n*=59), hybrid PKS-NRPS (*n*=25), RiPPS (*n*=76) and terpene (*n*=86). Network connections were established using a sequence similarity threshold of 0.3, the default cutoff for defining gene cluster families. This threshold ensures that nodes clustered together share substantial sequence and domain similarity. Of note, 220 nodes were identified as singletons, with 102 originating from Cuatro Ciénegas and 118 from Calakmul, highlighting a large fraction of BGCs unique to our isolates.

**Fig. 3.**
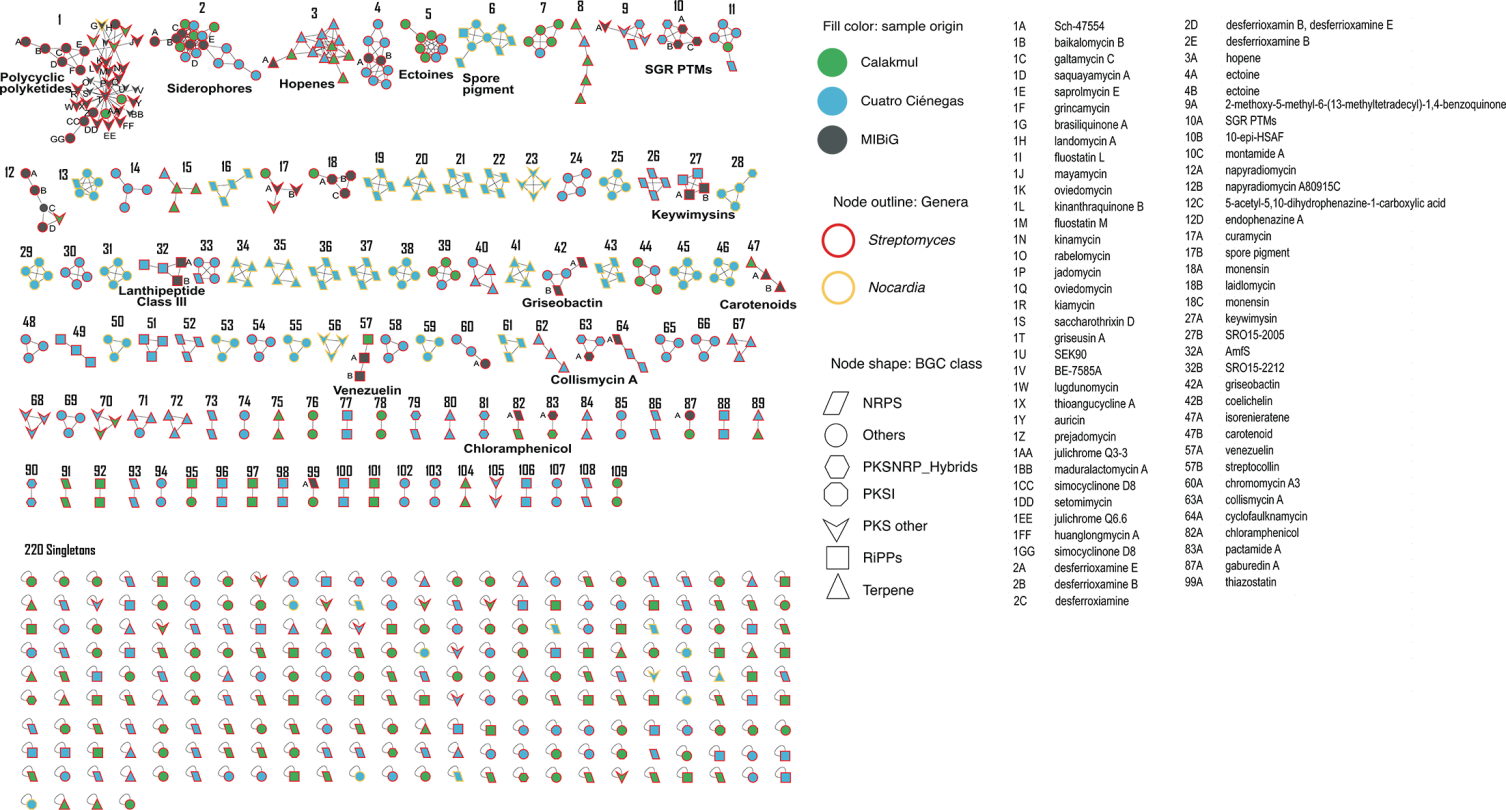
BiG-SCAPE network of BGCs from Cuatro Ciénegas Basin and Calakmul isolates. Each node represents a BGC. Node colour indicates the isolation source, while node outline colour indicates the genus. Node shape denotes the BGC class. Subnetworks are numbered. Each node from MIBiG is labelled with a letter. The adjacent list contains the known BGC from MIBiG. Singletons are included.

The results show that 20 subnetworks within the entire dataset are connected to a BGC from the MIBiG database. Specifically, 88.6% (*n*=497) of the BGCs were not associated with any known BGC. Subnetwork 1 includes polycyclic polyketide BGCs (auricin, BE-7585A, rabelomycin, dehydrorabelomycin, fluostatin, griseusin and setomimycin) (Fig. 3). Only five BGCs from our dataset fit into this subnetwork, including strains isolated from Calakmul. In addition, subnetwork 2 showed multiple strong matches to characterized siderophore BGCs, including desferrioxamine-related BGCs, reflecting both sequence similarity and conserved domain architecture. Twelve of our BGCs, mostly from CCB strains, were classified within this category (Fig. 3). Among the subnetworks most enriched in our isolates, the hopene-related cluster (subnetwork 3, Fig. 3) includes 12 nodes connected to the hopene BGC from *S. coelicolor* A3(2) (MIBiG accession BGC0000663). Interestingly, 27 exclusive subnetworks of *Nocardia* containing 107 of the total 119 BGCs were identified. This reflects the genus-specific repertoire of biosynthetic gene clusters between this bacterium and *Streptomyces*. Notably, only one BGC from *Nocardia* corresponds to the RiPP category, which may reflect a reduced need for competitive or antagonistic interactions in their native environment.

### Untargeted metabolomic and chemoinformatic analysis

We tested the potential of metabolite production for the strains isolated under laboratory conditions. We generated a GNPS molecular network for 34 microbial extracts, including media/solvent blanks. Strains CC208B and CC201A were not included in the analysis as they did not grow on ISP2 media. The network generated consisted of 4,675 parent ions containing MS² or fragmentation data, also referred to as nodes. A total of 3,443 nodes were singletons, indicating that their fragmentation patterns did not cluster with any other ions, suggesting a high degree of chemical diversity within the dataset (Fig. S12).

**Fig. 4.**
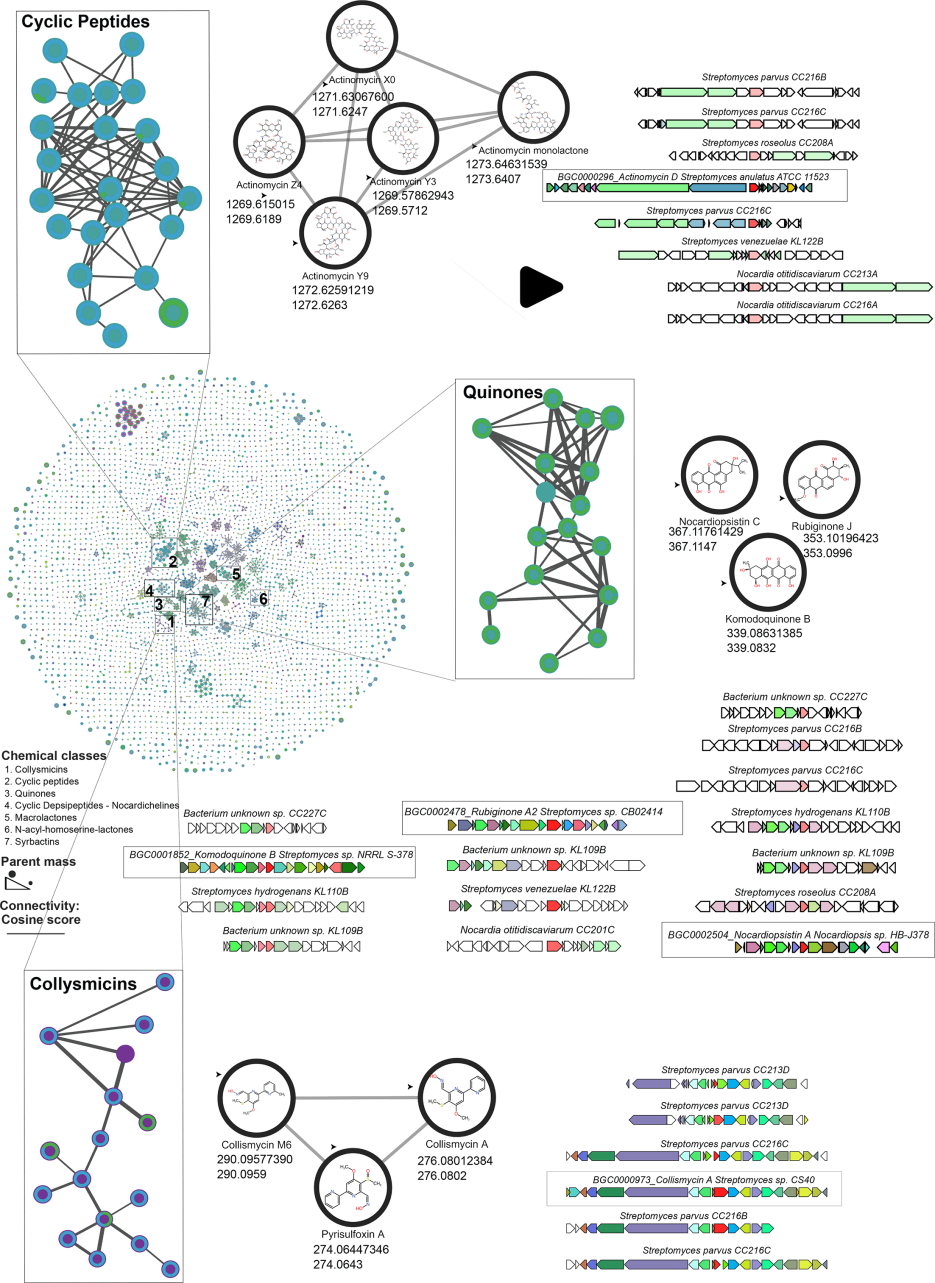
Molecular network of microbial metabolites with highlighted subnetworks and BGC linkages. The network shows metabolite features detected in microbial extracts, with nodes representing parent ions and edges indicating structural similarity based on cosine score. Seven subnetworks (1–7) are highlighted, some of which contain metabolites structurally characterized by SNAP-MS. Conserved biosynthetic gene clusters corresponding to these subnetworks are shown with synteny analysis using CORASON. Node colours indicate superclasses determined by the NP Classifier, and node size reflects parent mass. This integrated visualization links metabolite clusters with their putative BGCs and emphasizes both known and novel features identified in the dataset. Values under the molecules' names indicate theoretical (up) and experimental (down) mass.

The MolNetEnhancer workflow using NPClassifier [37] identified 188 putative chemical classes belonging to 59 chemical superclasses (Fig. 4). Nearly 30% of the detected parent ions could not be classified, further supporting the presence of chemically novel compounds. The most abundant superclass families were oligopeptides, small peptides, fatty amides and pseudoalkaloids, which collectively account for 39% of the total metabolite distribution. When adding the proportion of unclassified metabolites to the classified superclass, these compounds together represent 63% of the network, underscoring their dominance in the dataset.

In contrast, a smaller subset of the superclass, including macrolides and nucleosides, was present at 1–2% of the total distribution. Most detected superclasses were found at proportions below 1%, with 51 categories falling into this low-abundance group. These include rarer molecular classes such as tropolones, terphenyls and amino acid glycosides, some of which were detected at extremely low frequencies (~0.02%). Overall, our results should be interpreted as a descriptive overview of the chemical space captured under the culture conditions tested in our study, rather than a comprehensive representation of all possible metabolites produced by the strains.

### Natural product annotation via molecular networking and structural fingerprinting

We applied the SNAP-MS [40]. This approach allowed us to refine the assignment of subnetworks that were initially ambiguous or only partially annotated by GNPS, increasing confidence in the classification of several compound families. Using this approach, we annotated several antimycotic compounds. While GNPS correctly detected these compounds, MolNetEnhancer analysis using NPClassifier classified them as macrolides, raising doubts regarding their precise annotation. SNAP-MS confirmed that specific nodes within this subnetwork corresponded to antimycins, reinforcing our confidence in reclassifying the entire network as macrolactones (Fig. 4).

Similar cases included collismycin, several quinones such as rabelomycin and rhodomycinone, various *N*-acyl-homoserine-lactone derivatives, depsipeptides such as syrbactins, and cyclic peptides such as actinomycin. We also annotated compounds related to microcholine, mangromycin I and lactones, enabling the classification of subclusters within cyclic depsipeptides, macrocyclic polyketides and aliphatic lactones (Fig. 4). In each case, SNAP-MS provided crucial support in clarifying compound identities, ensuring more accurate classification within the molecular network.

Given the entire biosynthetic space and despite the advances in dereplication strategies, the putative annotations represent only a small fraction of the 561 BGCs detected across our isolates. A complete list of putatively annotated metabolites, obtained through GNPS spectral library matching and *in silico* structure annotation tools, is provided in Table S2. It should be noted that metabolite extraction was performed from the biomass fraction only, which may bias detection toward intracellular or non-secreted compounds. This methodological choice could explain why several of the putatively identified molecules (e.g. cytotoxic or DNA-damaging metabolites) are typically associated with intracellular functions. Characterization of secreted metabolites represents an important avenue for future work.

### Environmental origin significantly influences the metabolomic profiles of *Streptomyces* strains

**Fig. 5.**
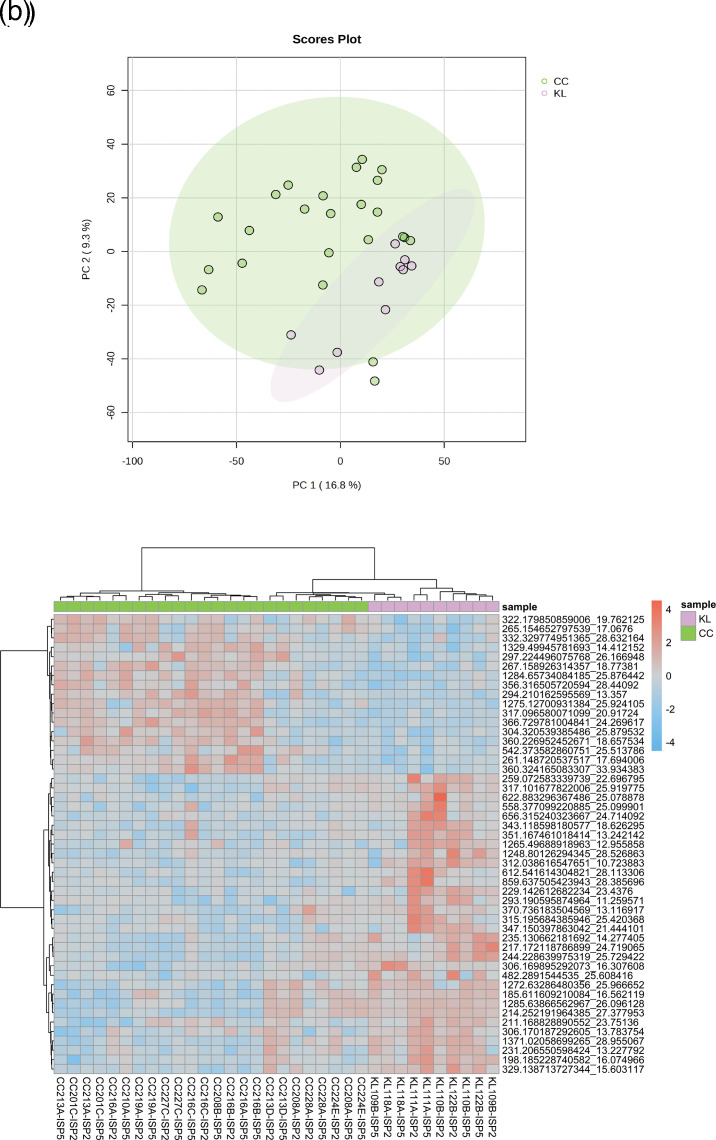
Untargeted metabolomic and chemoinformatic analysis. (**a**) PCA of normalized and filtered MS1 features quantified across all samples, grouped by sampling sites. (**b**) Heatmap of the top 50 MS1 of metabolites, ranked by Student’s t-test across sampling sites, highlighting differential abundance patterns.

Multivariate statistical analyses were conducted to evaluate the influence of the sample origin on the metabolic profiles of the isolates. Using normalized MS1 peak intensities, principal component analysis (PCA) and hierarchical clustering/HeatMap visualization analysis were performed using MetaboAnalyst. PCA results revealed distinct groupings of isolates according to their environmental origin, as reflected in the shaded areas of the plot (Fig. 5a). The heatmap (constructed using only the top 50 metabolites ranked by t-test significance) demonstrated clustering patterns consistent with the isolation sites, suggesting an important effect of geographic origin on the metabolomic composition (Fig. 5b). These results reinforce the observation that geographic origin influences the metabolomic composition of the isolates, although the partial overlap between groups suggests the presence of conserved metabolic features across environments. It is important to emphasize that these conclusions are exploratory, since the analysis is based on MS1 peak intensities rather than annotated metabolites, and thus does not allow assignment of chemical identities at this stage.

### Antifungal bioactivity evaluation of extracts against *C. albicans* SC5314

**Fig. 6.**
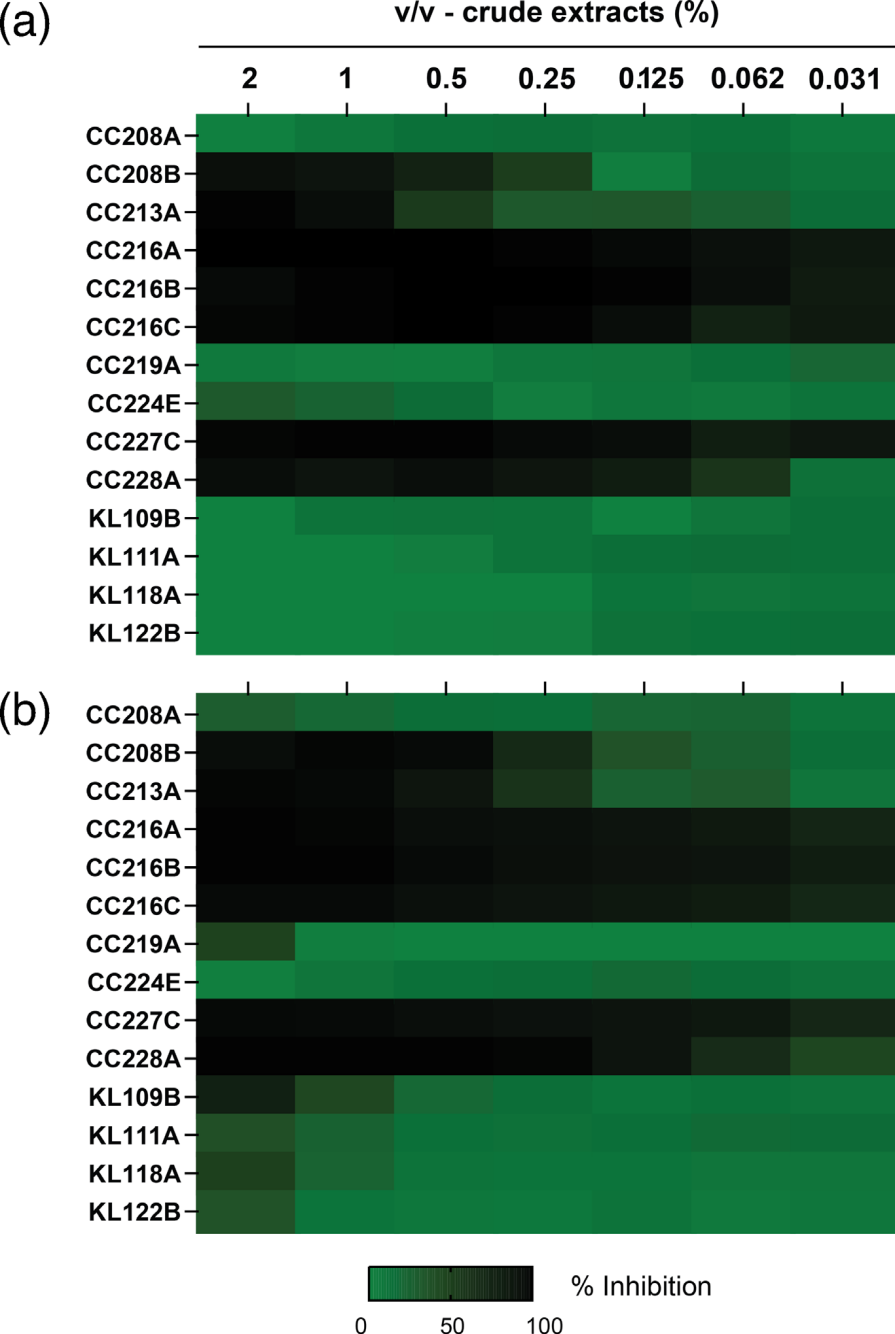
Antifungal bioactivity evaluation of 14 microbial crude extracts against *C. albicans* SC5314 expressed as % (v/v) of crude extracts. Heatmaps depict the inhibitory effects of the extracts on two growth modes of *C. albicans* SC5314: (**a**) planktonic growth and (**b**) biofilm formation. The colour scale reflects the relative inhibition, with darker shades indicating stronger antifungal activity.

Crude extracts from 14 bacterial strains were tested, in triplicate, for inhibition of *Candida albicans* SC5314 biofilm and planktonic growth, generating dose-response curves for each condition (Fig. 6) . Extracts from strains CC208B and CC213A exhibited substantial antibiofilm activity, reducing *C. albicans* SC5314 biofilm formation by up to 60% at concentrations as low as 0.25%. Extracts from a second group of strains: CC216A, CC216B, CC216C, CC227C and CC228A showed biofilm inhibition by at least 50% at the lowest concentration tested (0.031% v/v) and more than 90% at concentrations as low as 0.125%. Similar results were observed on planktonic growth. Extracts from strains CC208B and CC213A exhibited similar activity profiles, inhibiting planktonic growth by more than 90% at 1% treatments. Extracts from CC216A, CC216B, CC216C, CC227C and CC228A also inhibited more than 90% of planktonic growth at concentrations ranging from 0.25–0.062%. Interestingly, the minimum fungicidal concentration (MFC) of the extracts was also determined at 2% concentration for CC213A, CC216A, CC216B and CC227C, suggesting that the antimicrobial activity of these extracts is potentially due to fungicidal molecules.

Extracts from strains KL110B, CC213D, CC201C and CC210A could not be included in the assays because of insufficient yield and poor solubility in DMSO, which prevented proper testing. This limited solubility is likely due to the high polarity or complex resinous nature of these compounds, which are not readily soluble in this solvent.

### Linking MS² spectra to BGCs

The recently developed software framework NPOmix [48] was used to infer links between the MS² spectra of interest and their corresponding gene cluster families and BGCs. Utilizing a *k*-nearest neighbour algorithm, NPOmix establishes these associations independently of chemical class, integrating genomic and metabolomic data to facilitate the identification of specialized metabolites from bacterial cultures or environmental microbiomes. The pipeline relies on paired datasets with experimentally validated gene-mass spectral links, enabling automated connections between known spectra and their corresponding BGCs. Despite the promise of NPOmix, our attempts to establish confident BGC-MS² connections were inconclusive.

Alternatively, we conducted targeted bioinformatic analyses using CORe Analysis of Syntenic Orthologs (CORASON) [26,49], a tool designed to prioritize BGCs based on conserved gene architecture across genomes. CORASON was used to search for homologous gene clusters corresponding to structurally characterized compounds previously detected by SNAP-MS. In addition to collismycin and actinomycin D, which were previously annotated, we expanded our search to include antimycin, komodoquinone B, luminmycin A, nocardiopsistin A, rubiginone, rabelomycin and trichostatin RK.

Additionally, through this analysis, we identified highly similar BGCs associated with komodoquinone B, nocardiopsistin and rubiginone. Specifically, BGCs closely related to komodoquinone B were detected in *S. parvus* strains CC216C, CC216B and CC213D, as well as in strains CC227C, KL111A, KL109B and CC208B, all of which are putatively novel species. Similarly, BGCs closely related to nocardiopsistin were found in strains KL110B and the three strains CC208A, KL111A and KL109B, identified as potential novel species that share *S. roseolus* as their closest relative. Finally, rubiginone A2-like BGCs were detected in KL109B (a potential novel species), KL122B (identified as *Streptomyces venezuelae*), CC201C and CC216A, all classified as *Nocardia otitidiscaviarum* (Figs 4 and S1–S11).

## Discussion

The integration of metabolo-genomics datasets from *Streptomyces* and *Nocardia* strains isolated from CCB and the CBR offers a compelling view of how environmental pressures shape microbial secondary metabolism. Beyond presenting the first metabolo-genomic analysis of environmental isolates from two ecologically distinct Mexican biomes, our study underscores the strengths and persistent constraints of current genome mining frameworks, metabolomic approaches and cross-dataset integration.

A central finding of this study is the frequent disconnection between predicted BGCs and detected metabolites. For example, several metabolites observed in culture extracts lacked identifiable genomic origins, while certain BGCs had no corresponding mass spectrometry features. This mismatch suggests cryptic or silent biosynthetic pathways but also highlights practical issues such as genome fragmentation. We found that highly fragmented assemblies (high contig number) are associated with elevated proportions of incomplete BGCs and missed annotations, generating a bias in the estimation of taxonomic assignment and BiNI scores. Based on our experience, we recommend using high-quality assemblies, ideally ≤20 contigs for this type of analysis. Conversely, the absence of certain metabolite–BGC matches underscores deficiencies in current annotation algorithms and the need for improved reference databases.

Our work highlights that the expression of secondary metabolites is highly context-dependent. Media composition, culture conditions and extraction protocols critically influence metabolite production [50,51], reinforcing that condition-specific optimization is essential for fully unlocking the microbial chemical space. Such complexity must be accounted for during experimental design, especially when the goal is the dereplication of known compounds or the discovery of novel scaffolds with bioactive potential.

Taxonomic analyses provided additional insights into the novelty of several isolates. A polyphasic approach, employing ANI, dDDH and phylogenomic reconstruction, was essential for resolving the taxonomic status of strains falling below the 95% ANI species threshold. In such cases, dDDH values and phylogenetic topology were critical in suggesting that several isolates not only represent new species but may constitute previously unrecognized genera. These findings reaffirm the value of combining multiple genomic metrics, particularly for environmental isolates from underexplored or extreme habitats.

While our comparative metabolomic analyses revealed clustering of isolates by geographic origin, we recognize the limitations imposed by standard laboratory culturing. The strains analysed were isolated and grown using defined media (ISP2 and ISP5), which do not fully capture the complex physicochemical conditions of their native environments. Consequently, the observed metabolite profiles likely reflect a combination of ecological imprint and laboratory-induced expression. We acknowledge that cultivation-based methods inherently select for a subset of the native microbial community and that culture media composition can significantly modulate secondary metabolism. Therefore, any interpretation of environmental influence on metabolomic patterns should be made with caution, and future work integrating *in situ* or culture-independent approaches will be essential to refine these associations.

Ecological divergence was further reflected in the distribution and composition of BGCs between CCB and CBR strains. Isolates from CCB were enriched in BGCs associated with stress-adaptive compounds, such as melanin, ectoin, butyrolactones and non-ribosomal siderophores, metabolites likely selected under oligotrophic and physiochemically extreme conditions. These molecules confer ecological advantages such as UV protection [52], osmoregulation [53] and enhanced metal acquisition [54], reflecting evolutionary specialization to environmental stressors. In contrast, CBR-derived *Streptomyces* strains harboured a distinct repertoire, with abundant BGCs for phenazines and indoles, metabolites associated with microbial antagonism and interspecies competition. These patterns align with the ecological hypothesis that densely populated, biodiverse environments, such as tropical rainforests, exert selective pressure favouring the production of antimicrobial and antifungal compounds [55]. Consistent with this trend, the relative absence of ribosomally synthesized and post-translationally modified peptides in *Nocardia* from CCB suggests relaxed competition in the microbial community and a potential trade-off in favour of alternative survival strategies [56].

These environmental imprints on specialized metabolism were reflected in antifungal assays. Several isolates demonstrated potent inhibition of *C. albicans* SC5314 biofilm formation and planktonic growth at remarkably low extract concentrations. Notably, strains tentatively classified as novel species or closely related to *Nocardia otitidiscaviarum* and *Streptomyces parvus* exhibited up to fourfold greater inhibitory potency than the others. These results are currently being pursued through expanded bioassays targeting a broader panel of fungal pathogens, along with fractionation of bioactive extracts using advanced analytical platforms such as high-resolution mass spectrometry and NMR. Concurrent genome mining efforts aim to identify and prioritize BGCs potentially responsible for these antifungal effects, deepening our understanding of the genetic and chemical drivers of bioactivity.

The presence of bioactive molecules with antagonistic activity underscores the relevance of the isolates reported in this study. Collismycin exhibits cytotoxic and neuroprotective properties, particularly in oxidative stress models [57,58]. Rabelomycin, which is active against Gram-positive bacteria, also neutralizes reactive oxygen species, highlighting its antimicrobial and oxidative stress-related potential [59,60]. Rhodomycin serves as a key intermediate in anthracycline biosynthesis, enabling the production of antitumour compounds such as doxorubicin [61,62]. Actinomycin, which is known for its strong DNA-binding and transcription-blocking properties, remains a crucial tool in biological research and chemotherapy and has also shown antifungal properties [63,65].

Efforts to correlate MS² spectral features with specific BGCs leveraged tools such as NPOmix, antiSMASH and CORASON. While NPOmix employs a *k*-nearest neighbour approach to associate spectral features with gene cluster families independently of chemical class, the quality and coverage of existing reference datasets inherently limit its predictive power. However, in our study, genome mining using antiSMASH successfully identified the BGCs responsible for actinomycin D and collismycin biosynthesis. Complementarily, CORASON enabled the recognition of BGCs encoding komodoquinone, nocardiopsistin and rubiginone, demonstrating the utility of synteny- and ortholog-based approaches for targeted searches within large genomic datasets. Importantly, compounds such as rubiginone and derivatives have been reported to possess antifungal properties, including the inhibition of *Candida* biofilm formation and filamentation [66].

Our study demonstrates the advantages of using complementary tools to strengthen metabolo-genomic annotations. Nonetheless, the inability to retrieve hits for other target metabolites highlights persistent challenges in connecting chemical signatures with biosynthetic origins, particularly in the case of cryptic, fragmented or poorly characterized BGCs. Moving forward, enhancing the predictive accuracy of genome-metabolome interconnections will require iterative methodological refinement, integrating orthogonal data layers (e.g. transcriptomics and proteomics), and other reference databases. Crucially, such progress will rely on close collaboration between computational biologists, chemists and microbiologists to develop the next generation of integrative platforms capable of navigating the full complexity of microbial secondary metabolism.

Overall, this study demonstrates the importance of integrating genomics and metabolomics to explore the biosynthetic potential and ecological specialization of *Streptomyces* and *Nocardia* strains. By combining genome mining with untargeted metabolomic analyses and advanced annotation strategies, we annotated both known and previously undetected specialized metabolites of isolates from two relevant and contrasting Mexican biomes. Our study suggests distinctive ecological imprints on microbial specialized metabolism; however, interpretations must be made cautiously due to the influence of standardized laboratory culture conditions. Our findings reveal significant differences between predicted biosynthetic gene clusters and detected metabolites, underscoring the limitations of current tools and the context-dependent nature of secondary metabolite expression. Ultimately, this work promotes a systems-level integration of metabolomics, genomics, ecological and functional data to overcome current methodological limitations, while emphasizing the study of underexplored ecological niches to discover new biosynthetic clusters and natural products. Finally, our study contributes to demonstrating that advancing natural product discovery from microbial sources will rely not only on expanding multi-omics integration and improving reference datasets, but also on fostering interdisciplinary collaborations that bridge the gap between genomic potential and metabolite expression.

## Supplementary material

10.1099/mgen.0.001557 Uncited Supplementary Material 1.

10.1099/mgen.0.001557 Uncited Supplementary Material 2.

## Abbreviations

BGCs: biosynthetic gene clusters
BiNI: biosynthetic novelty index
CBR: Calakmul Biosphere Reserve
CCB: Cuatro Ciénegas Basin
CORASON: CORe Analysis of Syntenic Orthologs
dDDH: digital DNA–DNA hybridization
GNPS: Global Natural Products Social Molecular Networking
MFC: minimum fungicidal concentration
MSI: Metabolomics Standards Initiative
NPs: natural products
PBS: phosphate buffer solution
PCA: principal component analysis
QC: quality control
SNAP-MS: Structural Similarity Network Annotation Platform for Mass Spectrometry
TYGS: Type (Strain) Genome Server
YPD: yeast extract–peptone–dextrose
