# Corrigendum: Biodiscovery of Actinomycetota through metabolo-genomics reveals functional diversity across contrasting Mexican ecosystems

**DOI:** 10.1099/mgen.0.001641

**Published:** 2026-02-11

**Authors:** Lorena Rodríguez-Orduña, César Aguilar, Alan Gerardo Hernández-Melgar, Héctor F. Arocha Garza, Karina Verdel-Aranda, Augusto Vázquez Rodríguez, José L. López-Ribot, Aldo Moreno-Ulloa, Cuauhtémoc Licona-Cassani

**Affiliations:** 1 Tecnológico de Monterrey, Industrial Genomics Laboratory, Centro de Biotecnología FEMSA, Escuela de Ingeniería y Ciencias,, Monterrey, N.L., Mexico; 2 Biomedical Innovation Department, CICESE, Ensenada, B.C., Mexico; 3 Genesis Laboratory, Cuatro Ciénegas de Carranza, Coahuila, Mexico; 4 Tecnológico Nacional de México, Instituto Tecnológico de Chiná, Chiná, Campeche, Mexico; 5 Department of Molecular Microbiology and Immunology and South Texas Center for Emerging Infectious Diseases, The University of Texas at San Antonio, San Antonio, Texas, USA; 6 Instituto de Biotecnología, Universidad Nacional Autónoma de México, Cuernavaca, Morelos, 62210, Mexico; 7 Tecnológico de Monterrey, Integrative Biology Research Unit, The Institute for Obesity Research,, Monterrey, N.L., Mexico

In the published version of this article, the following sentence appears in the ‘Methods’ section under ‘LCMS² data processing and analysis’ :

"The results from FBMN, DEREPLICATOR+, CSI:FingerID, MolDiscovery, CANOPUS and NPClassifier were combined based on the previous work published by Osorio-Ramirez (https://www.mdpi.com/2072-6651/17/4/150) using a custom script derived from the MolNetEnhancer workflow [38], which is openly accessible at https://github.com/froz9/MolNetEnhancerMod."

The URL ‘https://www.mdpi.com/2072-6651/17/4/150’ was provided in place of a formal citation. This should have been included as a full reference. The article has now been updated to cite OsorioRamirez *et al*. appropriately, and the reference list has been amended accordingly.

