# Supplementary material for: Biodiscovery of Actinomycetota through metabolo-genomics reveals functional diversity across contrasting Mexican ecosystems
Source: Microb Genom. 2025 Oct 31;11(10):001557. doi: 10.1099/mgen.0.001557 (PMC13293302; doi:10.1099/mgen.0.001557)
Supplement: Uncited Supplementary Material 1. [file mgen-11-01557-s001.pdf]

Supporting information

Biodiscovery of actinomycetota through metabolo-genomics reveals functional diversity across contrasting Mexican ecosystems

Lorena Rodríguez-Orduña[1], César Aguilar [1], Alan Gerardo Hernández-Melgar[2], Hector F. Arocha Garza[3], Karina Verdel-Aranda[4], Augusto Vazquez Rodriguez[6], Jose L. Lopez-Ribot[6], Aldo Moreno-Ulloa[2], Cuauhtémoc Licona-Cassani[1,5]*

[1]Industrial Genomics Laboratory, Centro de Biotecnología FEMSA, Escuela de Ingeniería y Ciencias, Tecnológico de Monterrey, N.L. México.

[2]Biomedical Innovation Department, CICESE, Ensenada, B.C., México.

[3]Genesis.

[4]Tecnológico Nacional de México, Instituto Tecnológico de Chiná, Chiná, Campeche, México.

[5]Integrative Biology Research Unit, The Institute for Obesity Research, Tecnológico de Monterrey, N.L. México.

[6]Department of Molecular Microbiology and Immunology and South Texas Center for Emerging Infectious Diseases, The University of Texas at San Antonio, San Antonio, Texas, USA

***Corresponding author:** clicona@ibt.unam.mx

Table S1. Genome assembly statistics and quality metrics of the isolates

Strain	Contigs	N50	Total length (bp)	G+C content (%)	Coverage
KL110B	259	59,992	8,265,972	73,41	295
KL118A	250	68,044	9,080,607	71,51	22
CC216C	15	765,295	8,311,078	71,49	27,63
CC216B	187	101,506	8,235,849	71,61	36
CC213D	84	196,625	8,832,702	71,42	69
KL122B	18	800,383	7,863,776	72,61	47,63
CC201C	23	844,696	7,869,046	68,95	57,38
CC216A	52	286,61	8,105,468	68,87	298
CC213A	47	308,793	8,108,852	68,88	298
KL109B	38	636	9,342,629	71,04	34
KL111A	146	253,881	7,645,218	72,75	38,03
CC227C	47	399,138	7,582,608	68,97	298
CC208A	106	178,535	7,650,294	72,47	120
CC224E	15	1,179,171	7,119,332	73,59	295
CC228A	26	568,003	6,955,189	73,61	190
CC219A	334	79,53	7,406,592	73,40	297
CC210A	9	1,630,831	7,228,347	73,36	253
CC208B	1	6,927,693	6,927,693	73,40	50

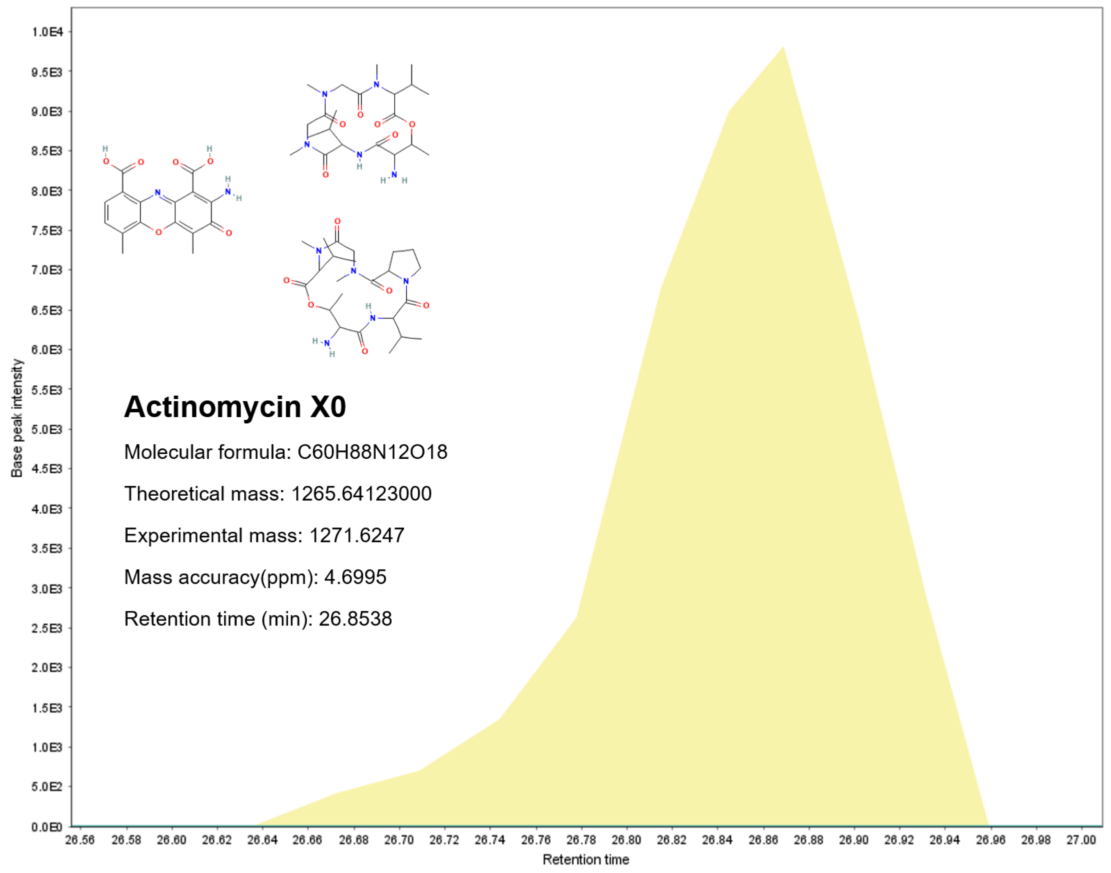

Figure S1. Extracted ion chromatogram (EIC) of Actinomycin X0. The chromatogram shows the underlying MS data including corresponding molecular formula, experimental and theoretical m/z, and retention time.

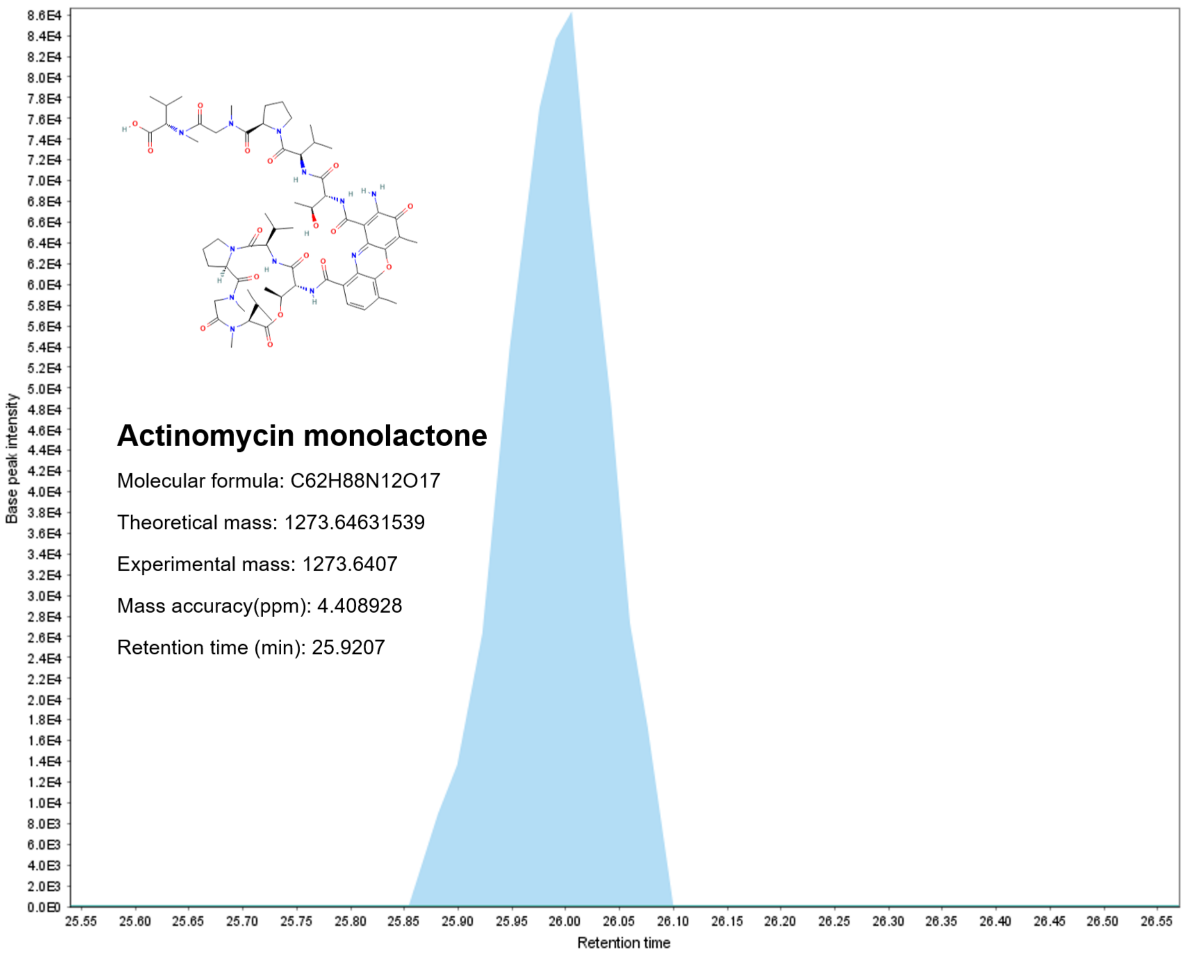

Figure S2. Extracted ion chromatogram (EIC) of Actinomycin monolactone. The chromatogram shows the underlying MS data including corresponding molecular formula, experimental and theoretical m/z, and retention time.

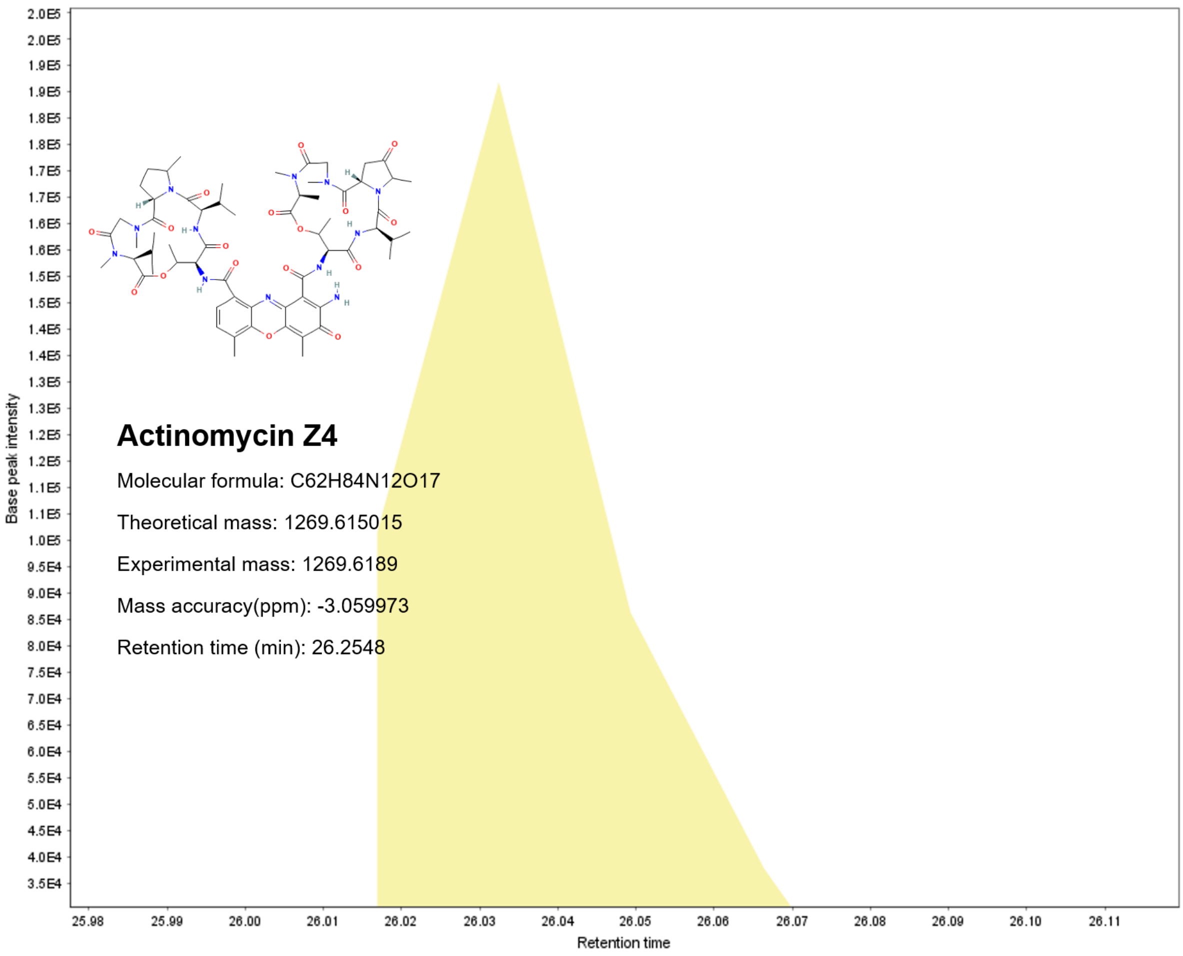

Figure S3. Extracted ion chromatogram (EIC) of Actinomycin Z4. The chromatogram shows the underlying MS data including corresponding molecular formula, experimental and theoretical m/z, and retention time.

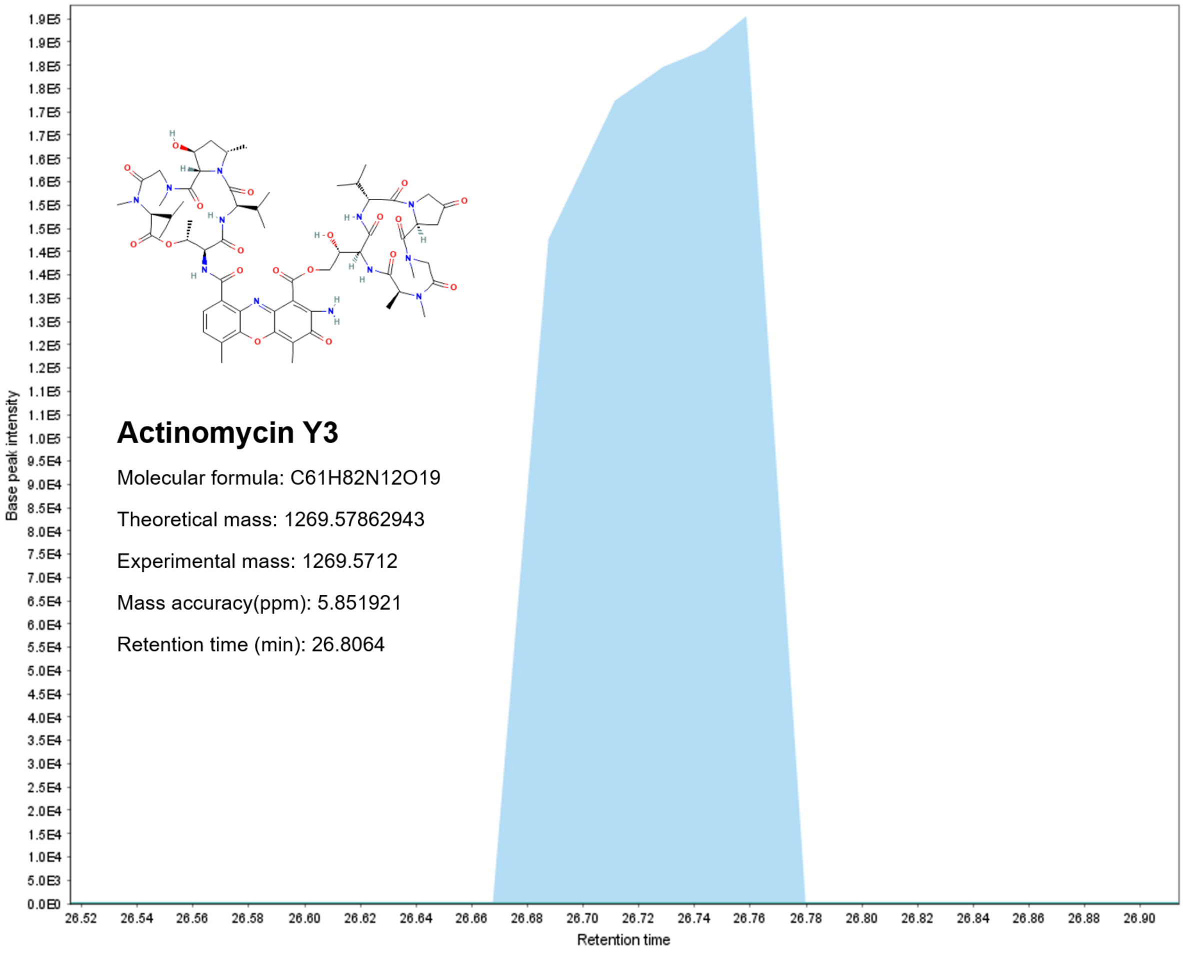

Figure S4. Extracted ion chromatogram (EIC) of Actinomycin Y3. The chromatogram shows the underlying MS data including corresponding molecular formula, experimental and theoretical m/z, and retention time.

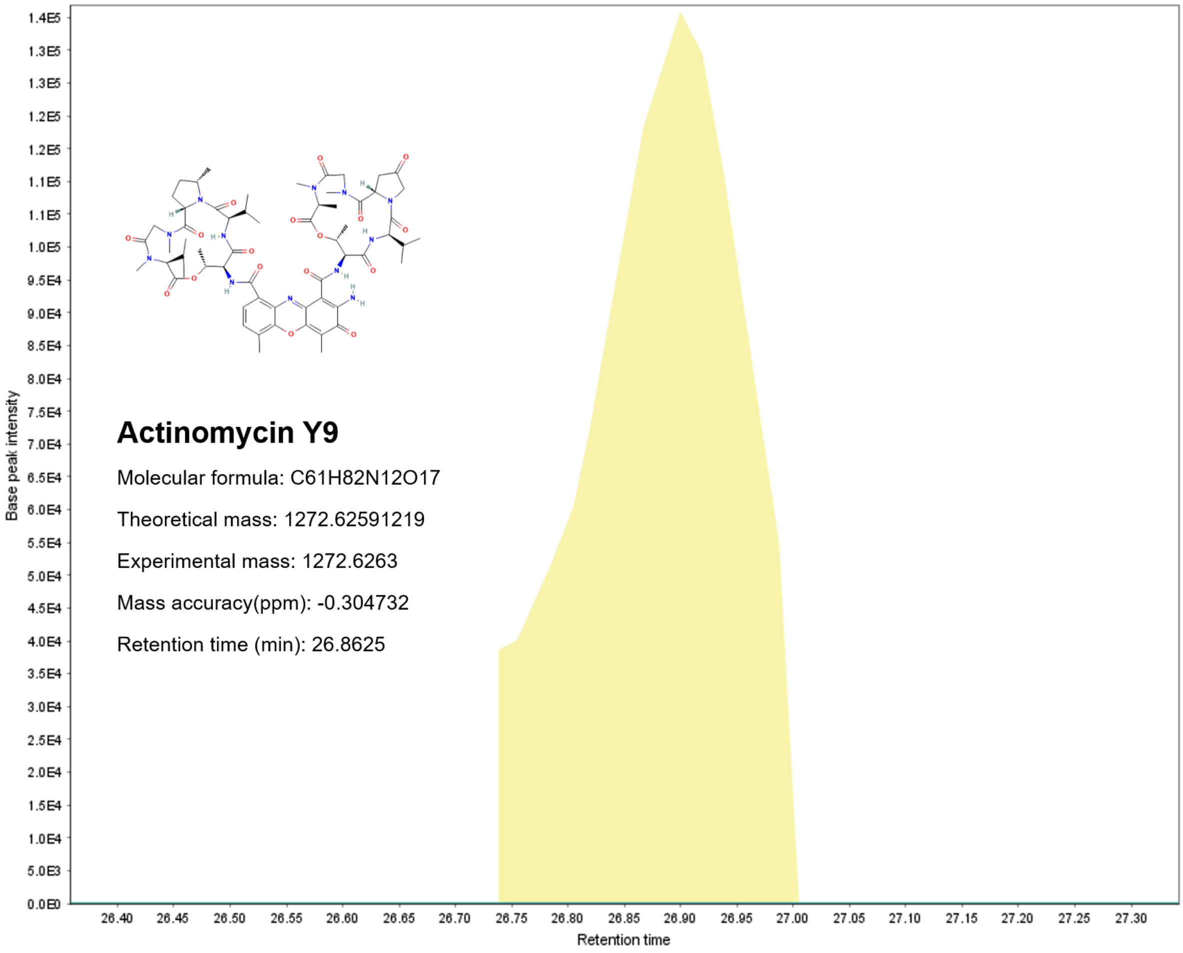

Figure S5. Extracted ion chromatogram (EIC) of Actinomycin Y9. The chromatogram shows the underlying MS data including corresponding molecular formula, experimental and theoretical m/z, and retention time.

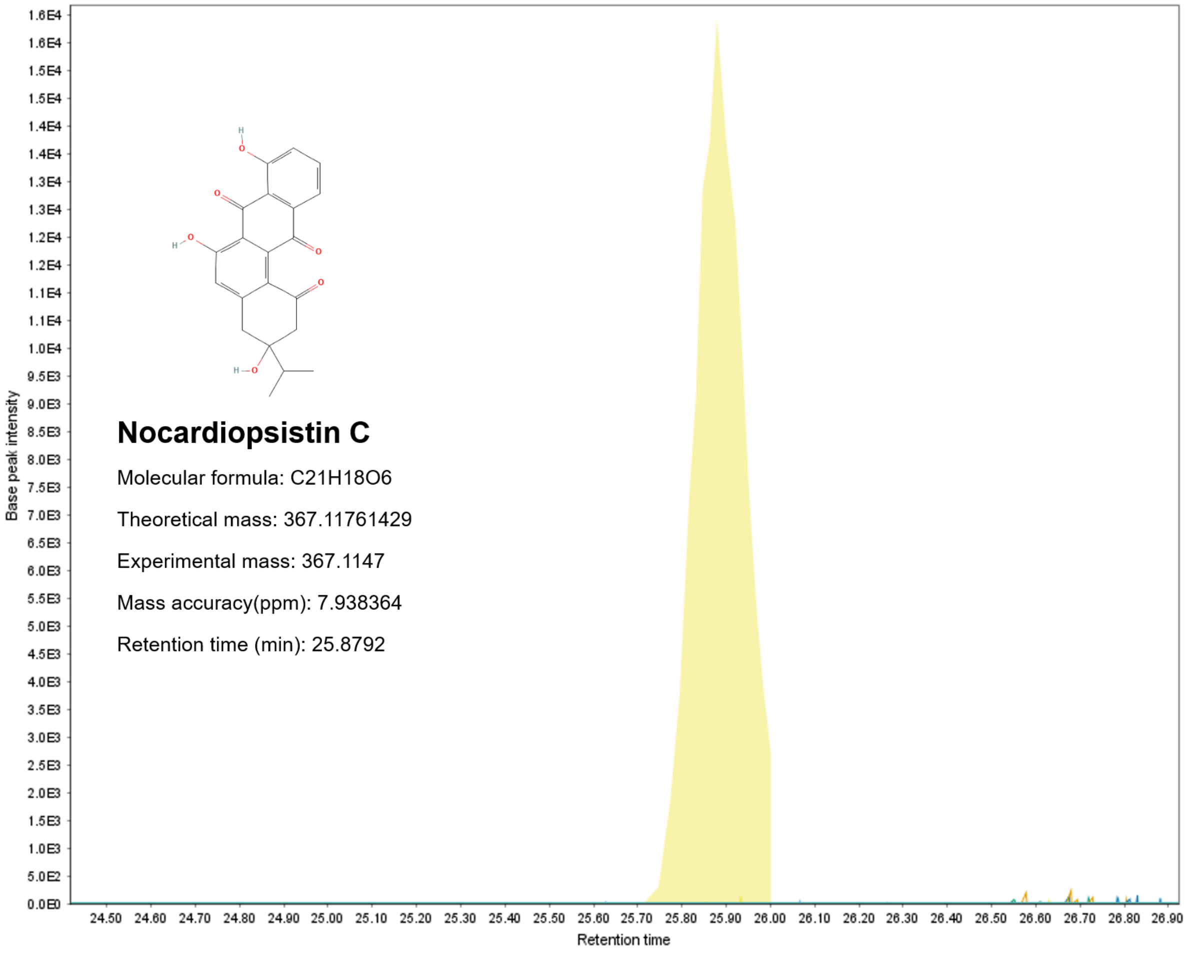

Figure S6. Extracted ion chromatogram (EIC) of Nocardiopsistin C. The chromatogram shows the underlying MS data including corresponding molecular formula, experimental and theoretical m/z, and retention time.

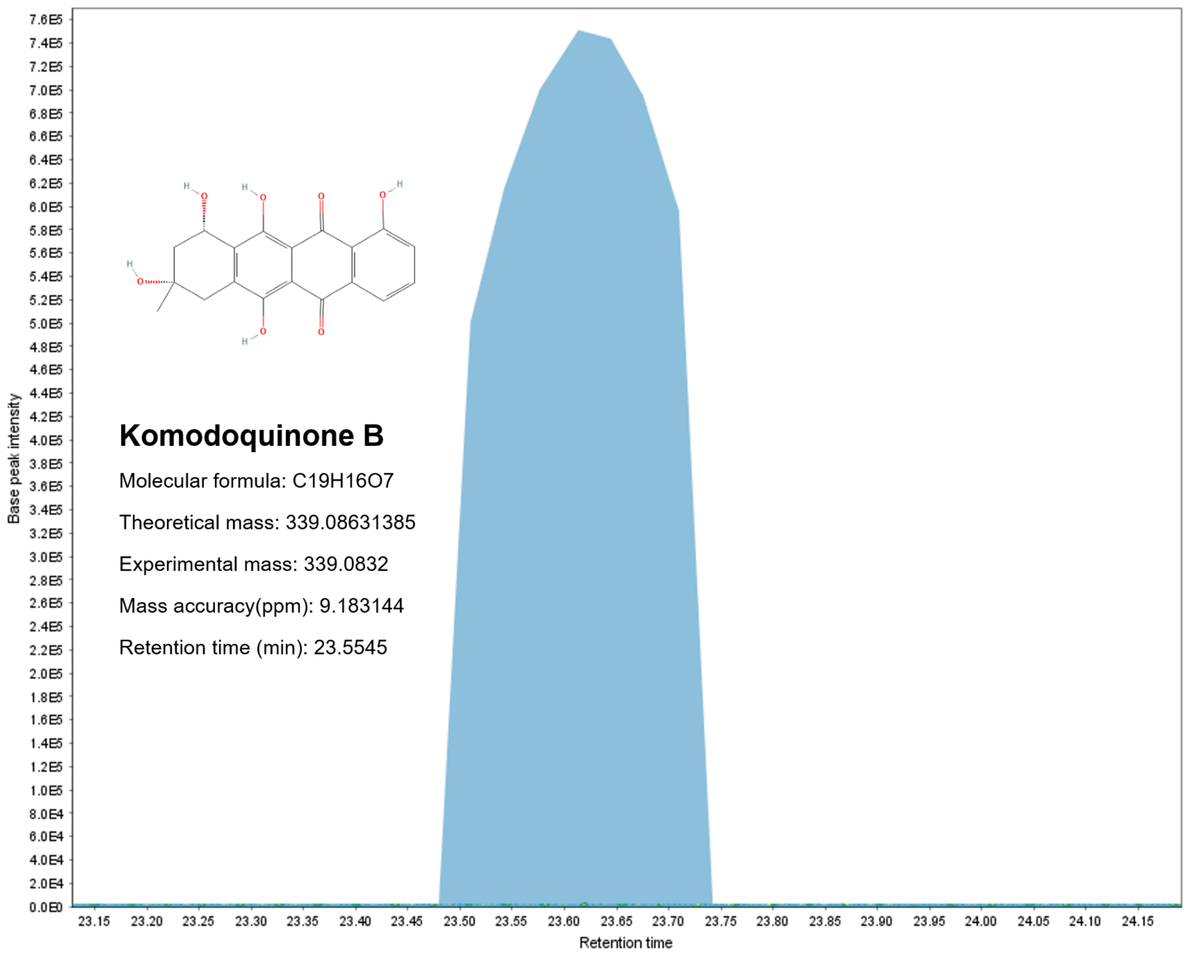

Figure S7. Extracted ion chromatogram (EIC) of Komodoquinone B. The chromatogram shows the underlying MS data including corresponding molecular formula, experimental and theoretical m/z, and retention time.

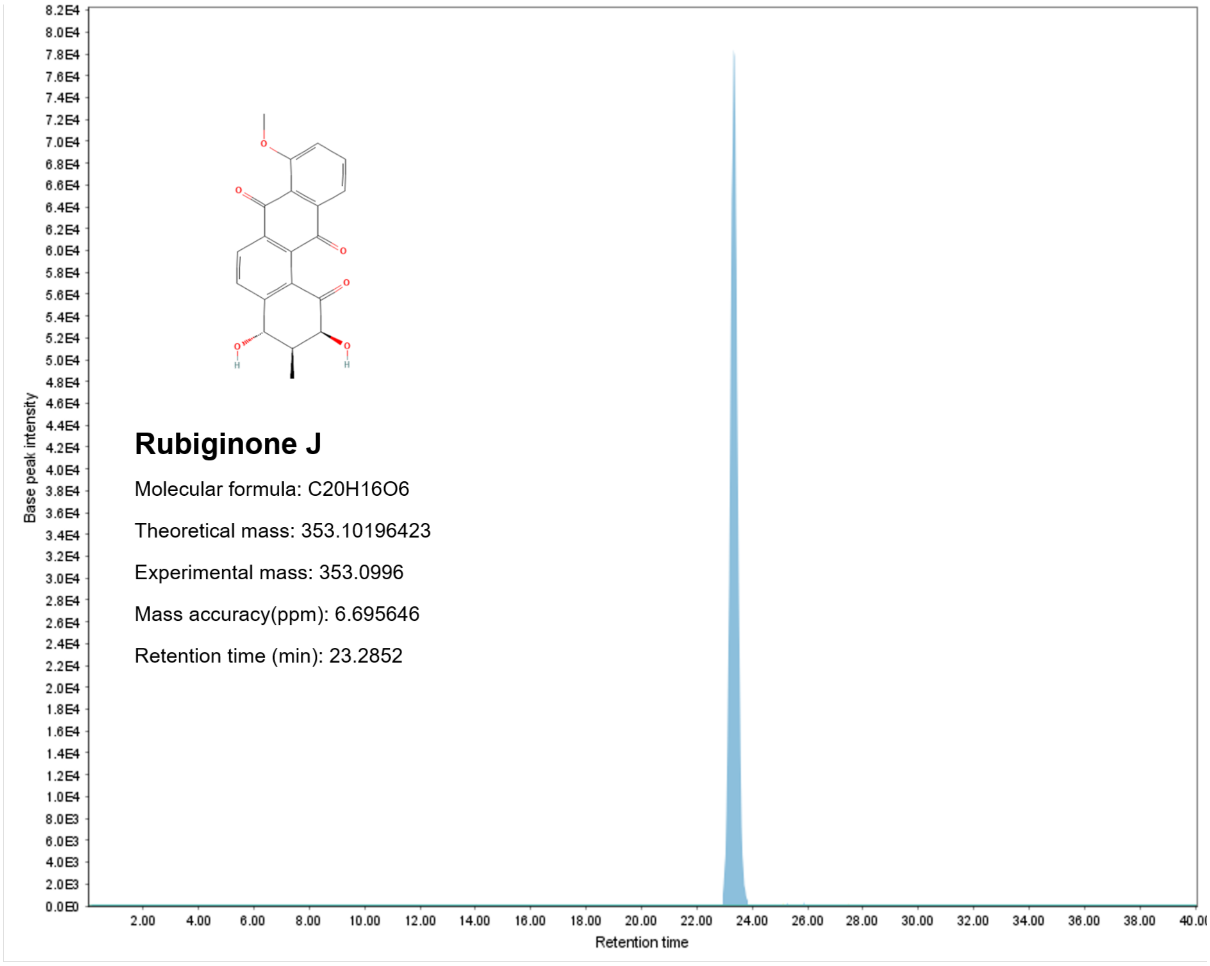

Figure S8. Extracted ion chromatogram (EIC) of Rubiginone J. The chromatogram shows the underlying MS data including corresponding molecular formula, experimental and theoretical m/z, and retention time.

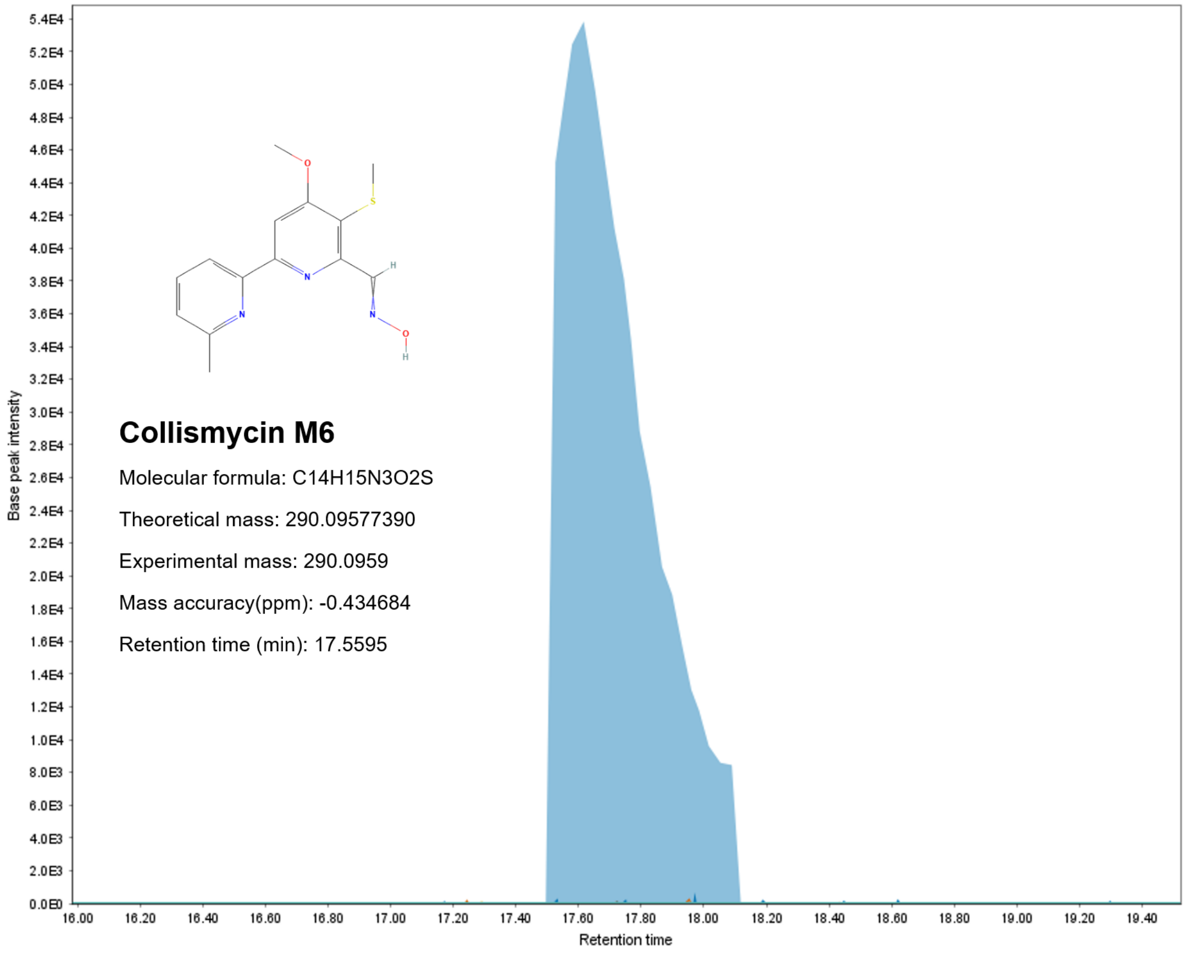

Figure S9. Extracted ion chromatogram (EIC) of Collismycin M6. The chromatogram shows the underlying MS data including corresponding molecular formula, experimental and theoretical m/z, and retention time.

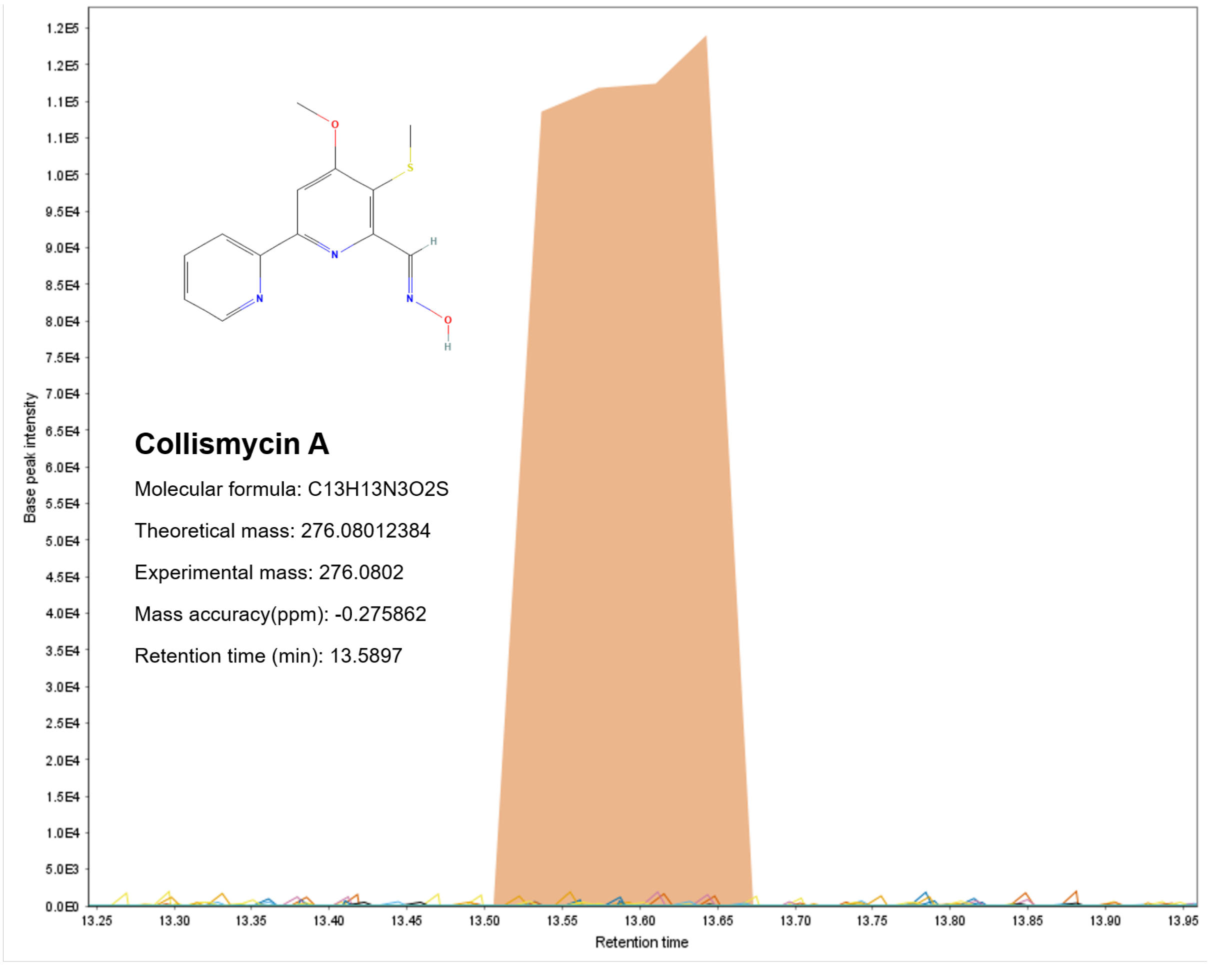

Figure S10. Extracted ion chromatogram (EIC) of Collismycin A. The chromatogram shows the underlying MS data including corresponding molecular formula, experimental and theoretical m/z, and retention time.

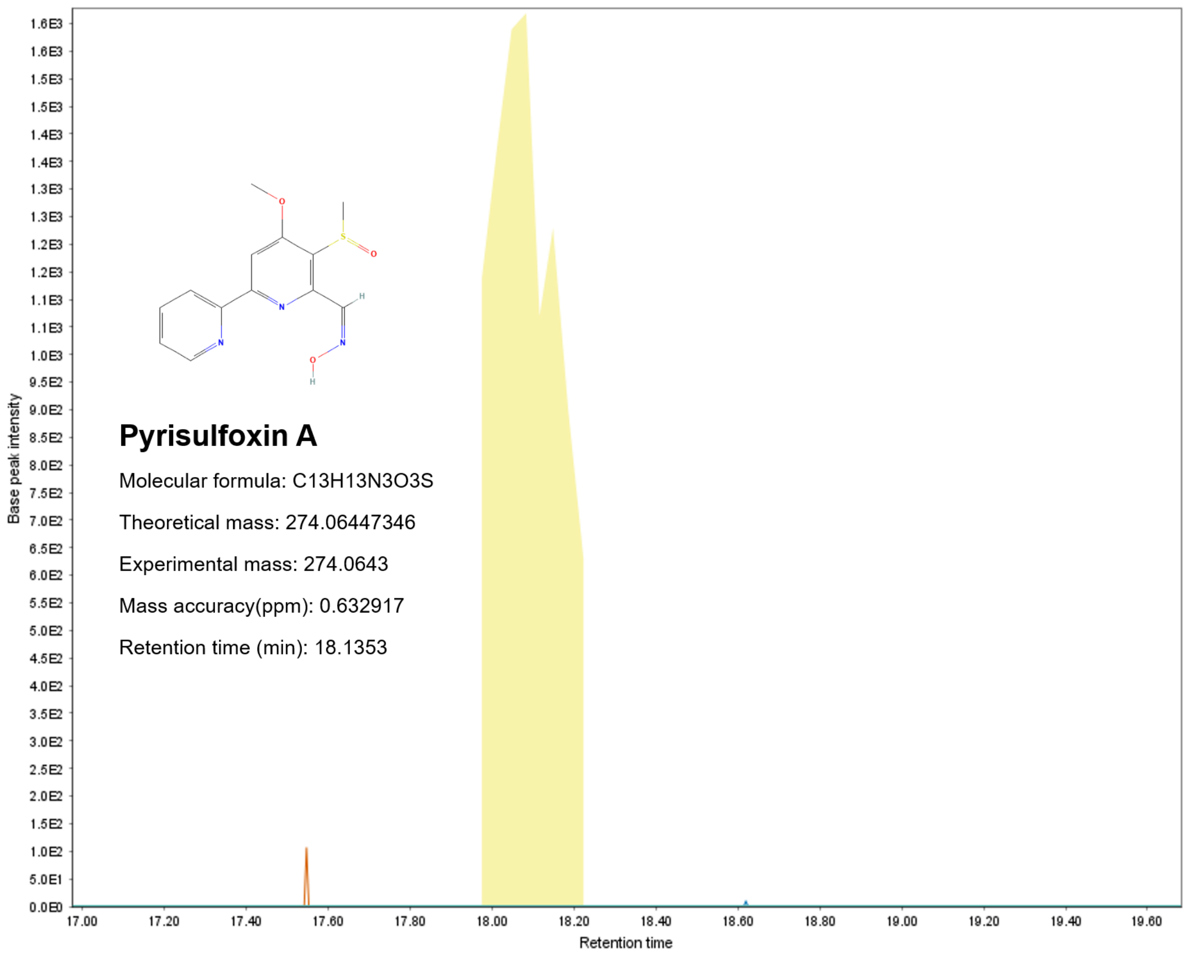

Figure S11. Extracted ion chromatogram (EIC) of Pyrisulfoxin A. The chromatogram shows the underlying MS data including corresponding molecular formula, experimental and theoretical m/z, and retention time.

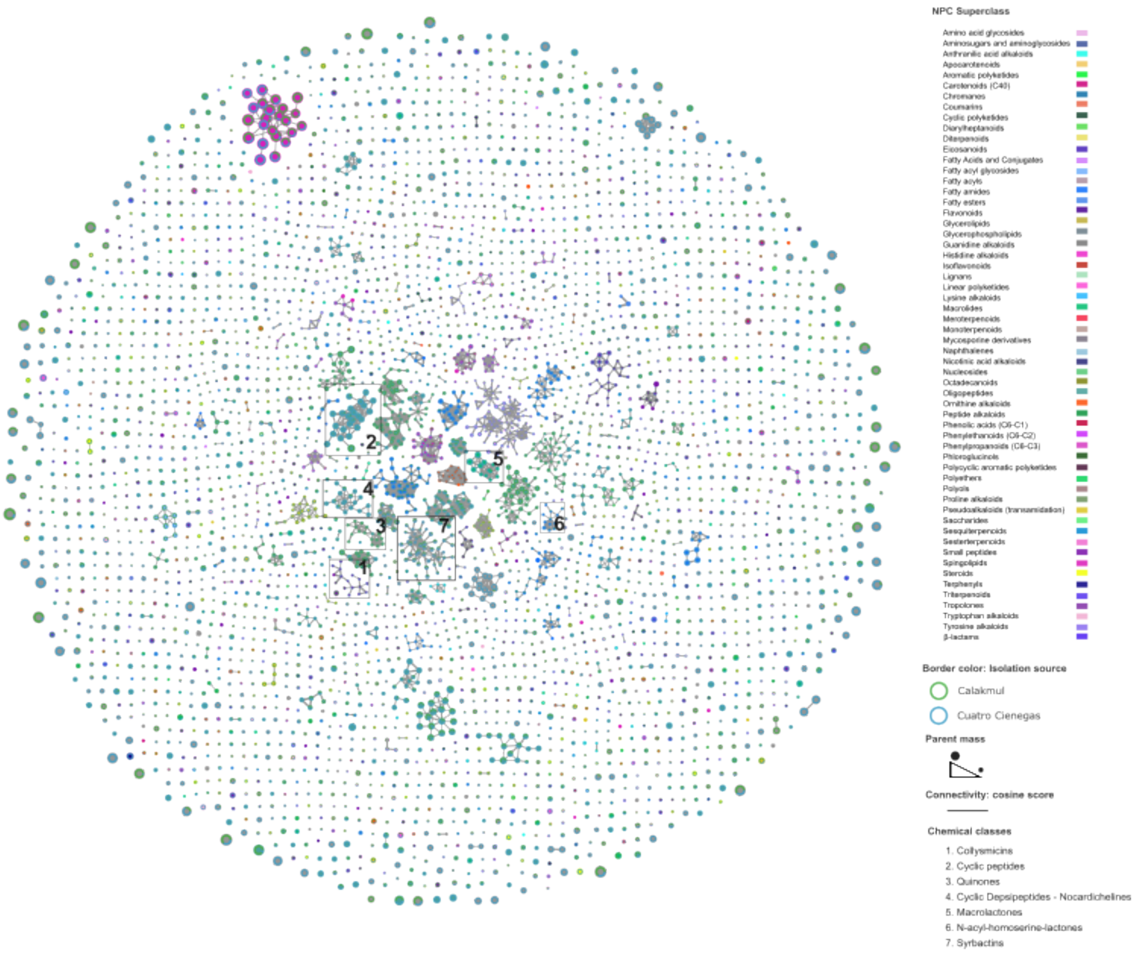

Figure S12. Molecular network of microbial metabolites with highlighted subnetworks. The network shows metabolite features detected in microbial extracts, with nodes representing parent ions and edges indicating structural similarity based on cosine score. Seven subnetworks (1–7) are highlighted, some of which contain metabolites structurally characterized by SNAP-MS. Node colors indicate superclasses determined by NP Classifier, and node size reflects parent mass.

Metabolomics data processing

Raw LC-MS² datasets in .d format were first converted to .mzXML using MSConvert from ProteoWizard (v3.0.24033) (Chambers et al., 2012) and subsequently processed in MZmine (v3.9.0) (Schmid et al., 2023). Processing steps included mass detection, chromatogram building and deconvolution using the ADAP algorithm, isotopic assignment, feature alignment, and gap filling. Three filtering criteria were applied: (i) features present in blank samples (culture media) were removed; (ii) only features observed in at least two aligned samples were retained; and (iii) features required a minimum of two isotopic peaks. The resulting quantification table, containing blank-filtered feature areas aligned across all samples, was exported as .csv and then imported into NormalyzerDE (v1.3.4) https://normalyzerde.immunoprot.lth.se/

(Willforss et al., 2019) for feature area normalization. Missing values were imputed using the Random Forest algorithm in MetImp (v1.2) (Yang et al., 2022) . For statistical analyses, the normalized and imputed dataset was submitted to MetaboAnalyst 4.0 (Chong et al., 2018) for univariate and multivariate analyses, including principal component analysis (PCA) and hierarchical clustering with heatmap visualization. Data was log-transformed. Features with a fold change ≥ ±1.5 and p-value <0.05 (Student's t-test) were considered differentially abundant. MS²-containing features were exported as .csv and .mgf files for metabolite annotation using GNPS (Wang et al., 2016), along with in-silico tools including MolDiscovery (L. Cao et al., 2020), CSI:FingerID (Dührkop et al., 2015), and DEREPLICATOR+ (Mohimani et al., 2018). Feature-Based Molecular Networking (FBMN) was carried out on GNPS with precursor and fragment ion mass tolerance set to 0.02 Da, a minimum cosine score of 0.6, and at least four shared fragment ions.

All tentative metabolite annotations from GNPS and in-silico prediction tools were classified into chemical classes using ClassyFire (Djoumbou Feunang et al., 2016). Outputs from FBMN, in-silico annotations, and ClassyFire were integrated via an in-house modified version of MolNetEnhancer (Ernst et al., 2019) available here: https://github.com/froz9/MolNetEnhancerMod. The resulting networks were visualized in Cytoscape v3.10.1 (Shannon et al., 2003) at the subclass level. DEREPLICATOR+ and MolDiscovery were run on GNPS with a precursor/fragment ion tolerance of 0.02 Da, maximum charge 1, and database AllDB (~720k compounds). MS² data were also analyzed in SIRIUS v5.8.5 (Dührkop et al., 2019) to determine molecular formula, structure, and chemical class, selecting compounds <860 Da. Common contaminants (e.g., plasticizers, silanones), and features with mass errors >15 ppm were filtered out. Metabolite annotations from GNPS and in-silico tools were reported as level 2 and 3, respectively, according to Metabolomics Standards Initiative criteria. When SMILES strings lacked IUPAC names, the SMILES-to-IUPAC Translator (STOUT) (Rajan et al., 2021) was used. Data processing, analysis, and visualization were conducted in R v4.3 and RStudio 2023.06.0, with tables generated using MetaboCoreUtils (Rainer et al., 2022), cheminf (Finch J, 2023), and ChemmineR (Y. Cao et al., 2008).

References

Cao, L., Guler, M., Tagirdzhanov, A., Lee, Y., Gurevich, A., & Mohimani, H. (2020). *MolDiscovery: Learning Mass Spectrometry Fragmentation of Small Molecules* (p. 2020.11.28.401943). bioRxiv. https://doi.org/10.1101/2020.11.28.401943

Cao, Y., Charisi, A., Cheng, L.-C., Jiang, T., & Girke, T. (2008). ChemmineR: A compound mining framework for R. *Bioinformatics*, *24*(15), 1733–1734. https://doi.org/10.1093/bioinformatics/btn307

Chambers, M. C., Maclean, B., Burke, R., Amodei, D., Ruderman, D. L., Neumann, S., Gatto, L., Fischer, B., Pratt, B., Egertson, J., Hoff, K., Kessner, D., Tasman, N., Shulman, N., Frewen, B., Baker, T. A., Brusniak, M.-Y., Paulse, C., Creasy, D., … Mallick, P. (2012). A cross-platform toolkit for mass spectrometry and proteomics. *Nature Biotechnology*, *30*(10), 918–920. https://doi.org/10.1038/nbt.2377

Chemoinformatics Tools For Annotating Untargeted ESI-MS Metabolomics Data. (n.d.). Retrieved September 3, 2025, from https://jasenfinch.github.io/cheminf/

Chong, J., Soufan, O., Li, C., Caraus, I., Li, S., Bourque, G., Wishart, D. S., & Xia, J. (2018). MetaboAnalyst 4.0: Towards more transparent and integrative metabolomics analysis. *Nucleic Acids Research*, *46*(W1), W486–W494. https://doi.org/10.1093/nar/gky310

Djoumbou Feunang, Y., Eisner, R., Knox, C., Chepelev, L., Hastings, J., Owen, G., Fahy, E., Steinbeck, C., Subramanian, S., Bolton, E., Greiner, R., & Wishart, D. S. (2016). ClassyFire: Automated chemical classification with a comprehensive, computable taxonomy. *Journal of Cheminformatics*, *8*(1), 61. https://doi.org/10.1186/s13321-016-0174-y

Dührkop, K., Fleischauer, M., Ludwig, M., Aksenov, A. A., Melnik, A. V., Meusel, M., Dorrestein, P. C., Rousu, J., & Böcker, S. (2019). SIRIUS 4: A rapid tool for turning tandem mass spectra into metabolite structure information. *Nature Methods*, *16*(4), 299–302. https://doi.org/10.1038/s41592-019-0344-8

Dührkop, K., Shen, H., Meusel, M., Rousu, J., & Böcker, S. (2015). Searching molecular structure databases with tandem mass spectra using CSI:FingerID. *Proceedings of the National Academy of Sciences of the United States of America*, *112*(41), 12580–12585. https://doi.org/10.1073/pnas.1509788112

Ernst, M., Kang, K. B., Caraballo-Rodríguez, A. M., Nothias, L.-F., Wandy, J., Chen, C., Wang, M., Rogers, S., Medema, M. H., Dorrestein, P. C., & van der Hooft, J. J. J. (2019).

MolNetEnhancer: Enhanced Molecular Networks by Integrating Metabolome Mining and Annotation Tools. *Metabolites*, *9*(7), Article 7. https://doi.org/10.3390/metabo9070144

Finch J (2023). cheminf: Chemoinformatics Tools For Annotating Untargeted ESI-MS Metabolomics Data. R package version 0.1.0, https://jasenfinch.github.io/cheminf/.

Mohimani, H., Gurevich, A., Shlemov, A., Mikheenko, A., Korobeynikov, A., Cao, L., Shcherbin, E., Nothias, L.-F., Dorrestein, P. C., & Pevzner, P. A. (2018). Dereplication of microbial metabolites through database search of mass spectra. *Nature Communications*, *9*(1), 4035. https://doi.org/10.1038/s41467-018-06082-8

Rainer, J., Vicini, A., Salzer, L., Stanstrup, J., Badia, J. M., Neumann, S., Stravs, M. A., Verri Hernandes, V., Gatto, L., Gibb, S., & Witting, M. (2022). A Modular and Expandable Ecosystem for Metabolomics Data Annotation in R. *Metabolites*, *12*(2), 173. https://doi.org/10.3390/metabo12020173

Rajan, K., Zielesny, A., & Steinbeck, C. (2021). STOUT: SMILES to IUPAC names using neural machine translation. *Journal of Cheminformatics*, *13*(1), 34. https://doi.org/10.1186/s13321-021-00512-4

Schmid, R., Heuckeroth, S., Korf, A., Smirnov, A., Myers, O., Dyrlund, T. S., Bushuiev, R., Murray, K. J., Hoffmann, N., Lu, M., Sarvepalli, A., Zhang, Z., Fleischauer, M., Dührkop, K., Wesner, M., Hoogstra, S. J., Rudt, E., Mokshyna, O., Brungs, C., … Pluskal, T. (2023). Integrative analysis of multimodal mass spectrometry data in MZmine 3. *Nature Biotechnology*, *41*(4), 447–449. https://doi.org/10.1038/s41587-023-01690-2

Shannon, P., Markiel, A., Ozier, O., Baliga, N. S., Wang, J. T., Ramage, D., Amin, N., Schwikowski, B., & Ideker, T. (2003). Cytoscape: A software environment for integrated models of biomolecular interaction networks. *Genome Research*, *13*(11), 2498–2504. https://doi.org/10.1101/gr.1239303

Wang, M., Carver, J. J., Phelan, V. V., Sanchez, L. M., Garg, N., Peng, Y., Nguyen, D. D., Watrous, J., Kapono, C. A., Luzzatto-Knaan, T., Porto, C., Bouslimani, A., Melnik, A. V., Meehan, M. J., Liu, W.-T., Crüsemann, M., Boudreau, P. D., Esquenazi, E., Sandoval-Calderón, M., … Bandeira, N. (2016). Sharing and community curation of mass spectrometry data with Global Natural Products Social Molecular Networking. *Nature Biotechnology*, *34*(8), 828–837. https://doi.org/10.1038/nbt.3597

Willforss, J., Chawade, A., & Levander, F. (2019). NormalyzerDE: Online Tool for Improved Normalization of Omics Expression Data and High-Sensitivity Differential Expression Analysis. *Journal of Proteome Research*, *18*(2), 732–740. https://doi.org/10.1021/acs.jproteome.8b00523

Yang, J., Sui, H., Jiao, R., Zhang, M., Zhao, X., Wang, L., Deng, W., & Liu, X. (2022). Random-Forest-Algorithm-Based Applications of the Basic Characteristics and Serum and Imaging Biomarkers to Diagnose Mild Cognitive Impairment. *Current Alzheimer Research*, *19*(1), 76–83. https://doi.org/10.2174/1567205019666220128120927
